# Genomic Surveillance of Circulating SARS-CoV-2 in South East Italy: A One-Year Retrospective Genetic Study

**DOI:** 10.3390/v13050731

**Published:** 2021-04-22

**Authors:** Loredana Capozzi, Angelica Bianco, Laura Del Sambro, Domenico Simone, Antonio Lippolis, Maria Notarnicola, Graziano Pesole, Lorenzo Pace, Domenico Galante, Antonio Parisi

**Affiliations:** 1 Istituto Zooprofilattico Sperimentale della Puglia e della Basilicata, Via Manfredonia, 20, 71121 Foggia, Italy; loredana.capozzi@izspb.it (L.C.); biancoange87@libero.it (A.B.); laura.delsambro@hotmail.it (L.D.S.); dome.simone@gmail.com (D.S.); lorenzo.pace@izspb.it (L.P.); domenico.galante@izspb.it (D.G.); 2 National Institute of Gastroenterology “S. de Bellis”, Institute of Research, Via Turi, 27, 70013 Castellana Grotte, Italy; antonio.lippolis@irccsdebellis.it (A.L.); maria.notarnicola@irccsdebellis.it (M.N.); 3 Institute of Biomembranes, Bioenergetics and Molecular Biotechnologies, CNR, Via Amendola 122/O, 70126 Bari, Italy; graziano.pesole@uniba.it

**Keywords:** COVID-19, SARS-CoV-2, genomic surveillance, whole genome sequencing, Nextstrain clade, Pangolin lineage, variants of concern

## Abstract

In order to provide insights into the evolutionary and epidemiological viral dynamics during the current COVID-19 pandemic in South Eastern Italy, a total of 298 genomes of SARS-CoV-2 strains collected in the Apulia and Basilicata regions, between March 2020 and January 2021, were sequenced. The genomic analysis performed on the draft genomes allowed us to assign the genetic clades and lineages of belonging to each sample and provide an overview of the main circulating viral variants. Our data showed the spread in Apulia and Basilicata of SARS-CoV-2 variants which have emerged during the second wave of infections and are being currently monitored worldwide for their increased transmission rate and their possible impact on vaccines and therapies. These results emphasize the importance of genome sequencing for the epidemiological surveillance of the new SARS-CoV-2 variants’ spread.

## 1. Introduction

Initially reported in mid-December 2019 in Wuhan, China, the newly emerged Severe Acute Respiratory Syndrome Coronavirus 2 (SARS-CoV-2) was identified as the cause of an outbreak of a severe respiratory infection, namely, Coronavirus Disease 2019 [1] (COVID-19).

Although Chinese authorities implemented strict quarantine measures in Wuhan and surrounding areas, this single-stranded RNA beta-coronavirus rapidly spread over 224 countries in one year. Up to 22 January 2021, there have been 95,612,831 confirmed cases of COVID-19 and 2,066,176 deaths, reported to the WHO [2]. The World Health Organization declared a COVID-19 pandemic on 11 March 2020.

After the first detection of SARS-CoV-2 in Italy in January 2020 [3] in two Chinese tourists [4,5], there has been an intense viral circulation which mainly concerned Northern Italy [6,7] and, later, all the other Italian regions. To date, Italy has one of the highest rates of SARS-CoV-2 infection among the developed countries, with 4047 cases per 100,000 people and a significant case/fatality ratio of about 3.5% (data from https://coronavirus.jhu.edu/map.html [8], accessed: 22 January 2021).

From a public health perspective, real-time whole-genome sequencing (WGS) of SARS-CoV-2 enables the tracking of spread patterns through genomic epidemiology and more insights into the pathogenesis and virulence of this virus by means of comparative genomic analysis; it may also provide support for the development of targeted vaccines. Furthermore, the assessment of the genetic diversity of SARS-CoV-2 has a crucial role in expanding our knowledge of the virus, in order to develop effective prevention and containment strategies.

The naturally expanding genetic diversity of SARS-CoV-2 has brought up the need for real-time tracking of the pathogen evolution, leading several scientific research groups to introduce a classification system for major circulating viral clades. The GISAID (Global Initiative on Sharing All Influenza Data) nomenclature system, developed by Sebastian Maurer-Stroh et al., is based on marker mutations within six high-level phylogenetic groupings, from the early split of S and, to the further evolution of L into V and G and later of G into GH and GR [9]. The definition of more detailed lineages by the Phylogenetic Assignment of Named Global Outbreak LINeages (PANGOLIN) tool [10] further helps to understand the patterns and determinants of SARS-CoV-2 global spread, expanding the GISAID clade definitions. This software tool describes lineages as a set of sequences observed in a geographically distinct region with evidence of ongoing transmission in that area. PANGOLIN provides a combination of genetic and epidemiological support for this nomenclature system, since phylogenetic information and the variety of metadata associated with a sequence contribute to the definition of lineages.

An additional classification effort has been provided by Nextclade [11], which initially employed a year-letter nomenclature to facilitate the discussion of large-scale diversity patterns of SARS-CoV-2 and labeled genetically well-defined clades that have achieved significant frequency and geographic spread. In the year-letter scheme, years are used to make immediate reference to the pandemic period in scientific discussions. This strategy allows to define a novel clade when it reaches >20% global frequency for more than 2 months.

Nextclade provides a classification, which supports the PANGOLIN lineage nomenclature system, based on non-synonymous amino acid mutations that characterize the two clades that emerged in 2019 [11] and the nine clades identified during 2020 [12].

Pangolin is a dynamic nomenclature system that assists the evolving genomic epidemiology of SARS-CoV-2: lineages provide a deeper level of classification and allow a subtyping of genomes belonging to the same clade. Often, the same lineage is assigned to genomes belonging to different clades.

Despite the deluge of epidemiologic data, to date there is still limited availability of shared genetic information of SARS-CoV-2 variants circulating in Italy: viral genomes submitted to public repositories represent about 0.1% of registered cases, leading to a potential surveillance bias due to delayed or underrepresented sequencing data from some areas.

This study aimed to perform whole-genome sequencing of 298 genomes sampled in the South Eastern Italian regions of Apulia and Basilicata and to provide a ‘real-time’ overview of the viral genotypes circulating in this geographical area. Genetic analysis of the sequences obtained and submitted to GISAID revealed the emergence of new lineages not yet widespread in Italy. These emerging variants have been detected since September 2020 and were most likely introduced during the summer holiday season.

This survey confirms the need to perform timely genome sequencing of a significant and representative selection of isolates, in order to develop targeted containment measures against known variants as well as new emerging variants of concern [13].

## 2. Materials and Methods

### 2.1. Specimen Collection and Testing

Clinical samples were collected between 31 March 2020 and 11 January 2021, from the Apulia and Basilicata regions. Nasopharyngeal swab specimens were stored in either phosphate-buffered saline (PBS) or viral transport medium COPAN UTM^®^ Universal Transport Medium (COPAN Diagnostics, Inc., Murrieta, CA, USA). Some nasopharyngeal swabs were subjected to isolation tests on Vero E6 cells; for these samples, RNA was isolated from cell culture supernatants. Viral RNA was purified using the QIAamp Viral RNA Mini kit (Qiagen, Hilden, Germany). The presence of SARS-CoV-2 was assessed by a multiplex real-time reverse-transcription polymerase chain reaction (rRT-PCR) test using Gene Finder™ COVID-19 Plus Real Amp Kit (OSANG Healthcare Co., Ltd., Gyeonggi-do, Korea). Real-time PCR was performed on an Applied Biosystems 7500 Real-Time PCR System (software version 2.3). Middle-high viral load samples (Ct < 30 for E gene) were selected for genome sequencing.

### 2.2. cDNA Synthesis and Viral Genome Amplification

cDNA synthesis was performed using Luna Script RT Super Mix Kit (New England Biolabs, Ipswich, MA, USA). The reaction mixture had a volume of 20 μL including 4 μL of 5× Luna Script RT Super Mix, 10 μL of purified viral RNA template, and 6 μL of distilled water, according to the manufacturer’s instructions. The synthesized cDNAs were diluted with nuclease-free water and used as templates for direct amplification performed in multiplexed PCR reactions, to generate ~400 bp amplicons tiled across the genome. The multiplex primer set, consisting of two non-overlapping primer pools, was provided by the ARTIC Network (V3 nCov-2019 primers) (ARTIC primer set [14]). PCR amplification was carried out using Q5 Hot Start High-Fidelity 2X Master Mix (New England Biolabs, Ipswich, MA, USA) with 6 μL of cDNA and 3.6 µl of V3 primer pool per a 25 µL reaction. Two separate reactions were carried out for each primer pool, respectively, primer pool 1 (10 µM) and 2 (10 µM). A two-step PCR program was used with an initial step of 98 °C for 30 s, then 35 cycles of 98 °C for 15 s followed by five minutes at 65 °C.

### 2.3. Library Preparation and Whole-Genome Sequencing

The genomic library preparation approach was adapted from the ARTIC (Advancing Real-Time Infection Control) V3 Network protocol and Illumina Nextera DNA Flex Library protocol (Illumina, San Diego, CA, USA).

Amplicons from both primer pools were combined and purified with a 1× volume of Ampure XP beads (Beckman Coulter, CA, USA). Approximately 300 ng of each purified sample of multiplexed PCR amplicons obtained was used for library preparation with Nextera DNA Flex sample preparation kit (Illumina), according to the manufacturer’s instructions [15]. Sequencing was performed on the Illumina MiSeq platform (Illumina) using the MiSeq Reagent Kit v2, 2 × 250 paired-end cycles.

### 2.4. Sequence Data Analysis

The paired-end raw reads were quality-filtered and trimmed using Trimmomatic v0.38 [16]. De novo genome assembly and scaffolding were performed with SPAdes v. 3.12 [17]. Quality check of genome assemblies was performed by aligning the filtered reads to the SARS-CoV-2 reference genome (GenBank: NC_045512.2) using Bowtie2 v2.3.4 [18]. Sequence alignments were converted to binary alignments (BAM format) and sorted using SAM-tools version 1.3.1. Read alignments were evaluated with QualiMap [19] (results are provided in Supplementary Table S2). Two hundred and ninety-eight consensus genome sequences passed the quality control assessment by Nextclade, whose parameters include missing data, mixed sites, private mutations, and mutation clusters, and were deposited in the GISAID database (https://www.gisaid.org/, accessed: 28 January 2021), with accession numbers provided in Supplementary Table S1.

### 2.5. Phylogenetic Analysis

**Figure 1:**
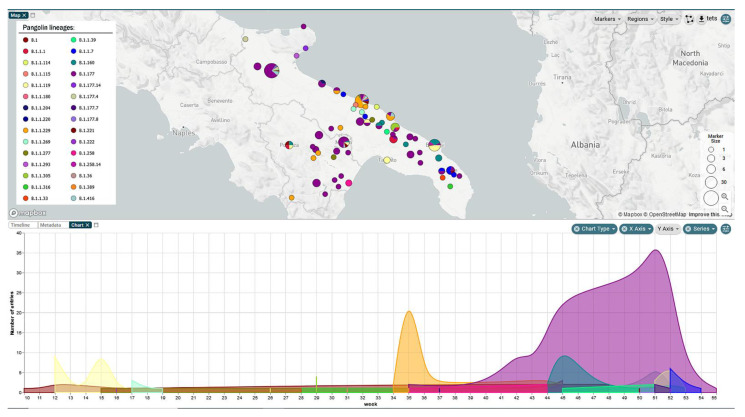
Pangolin lineages detected in Apulia and Basilicata regions, from March 2020 to January 2021.

The lineages of the reconstructed genomes were predicted with Pangolin [10], allowing for a geographical classification of circulating viral genomes. A dataset based on the clustering of SARS-CoV-2 genomes (passing quality filters) collected in Apulia and Basilicata was generated, with associated data including location, isolation date, and predicted Pangolin lineages. Microreact [20] was used to visualize the results of this phylogeographic analysis (Figure 1).

**Figure 2:**
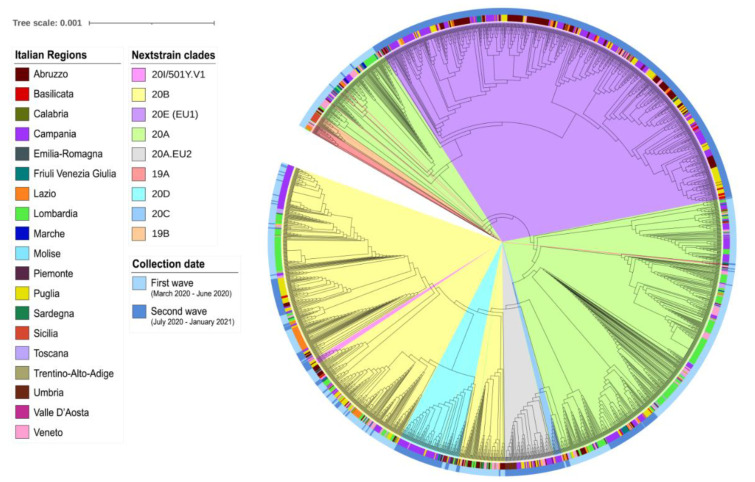
Phylogenetic tree showing the relationship between the 2244 Italian SARS-Cov-2 genome sequences. The tree was inferred with the approximate maximum likelihood method as implemented by FastTree v2.1 [21] and decorated with iTOL. Nextstrain clades are highlighted in pastel colors. The Italian region of sample collection is shown across the inner colored strip, the outer circle displays the sampling date, sorted into first wave and second wave. A newick (plain text) version of the phylogenetic tree, with branch lengths and support values at nodes, is reported in Supplementary File 2.

The Nextclade tool was used for isolate clades definition, mutation calling, and phylogenetic analyses (Nextstrain, Nextclade: https://clades.nextstrain.org/, accessed: 28 January 2021). For the phylogenetic analysis, all the publicly available SARS-CoV-2 genome sequences submitted from Italy starting from January 2020 were downloaded from GISAID on 25 January 2021. Sequences with <1% Ns were considered for subsequent analyses, thus resulting in a dataset of 1946 full genome sequences. Phylogenetic analysis was performed by building a multiple alignment of all the 2244 genome sequences using mafft v7.475 with the options—6 merpair–keeplength–addfragments. The resulting multiple alignment was used to build an approximate maximum likelihood tree with FastTree v2.1 [21] using a generalized time-reversible (GTR) model. The SARS-CoV-2 reference sequence (GenBank: NC_045512.2) was employed as outgroup. The GTR model was selected as the best-fit model of evolution upon evaluation with Model-Test-NG [22], based on Bayesian information criterion values. The phylogenetic tree is shown in Figure 2, while Supplementary File 2 reports a newick (plain text) version with branch lengths and support values at nodes.

### 2.6. Ethical Statement

Ethical approval was not provided for this study on human participants because the samples were collected during the last 3 years. After diagnostic routine, strains resulted from the biological material were stored to be processed for further analysis. No personal data or any other information than the type of material and the result of routine microbiology analysis were collected from each specimen, inhibiting any correlations of these fully anonymized samples with the respective patients. Thus, according to national regulations and the institutional rules for Good Scientific Practice, the requirement for submission to an ethical committee and for obtaining patients’ informed consent was waived. Written informed consent for participation was not required for this study in accordance with the national legislation and the institutional requirements.

## 3. Results

**Figure 3:**
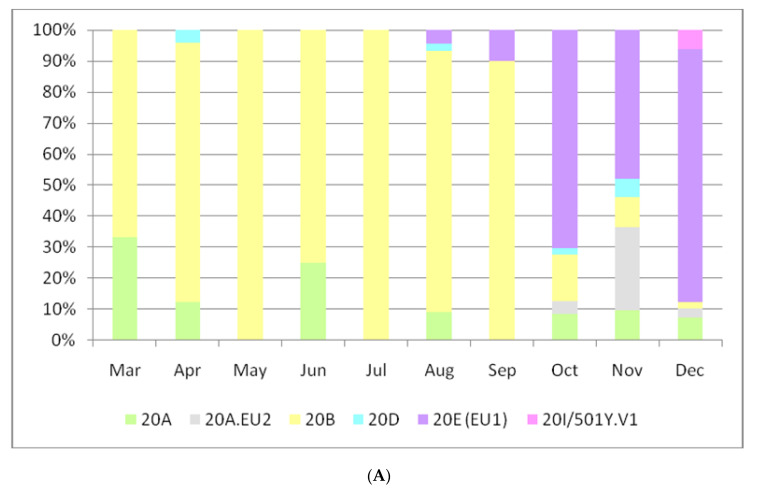

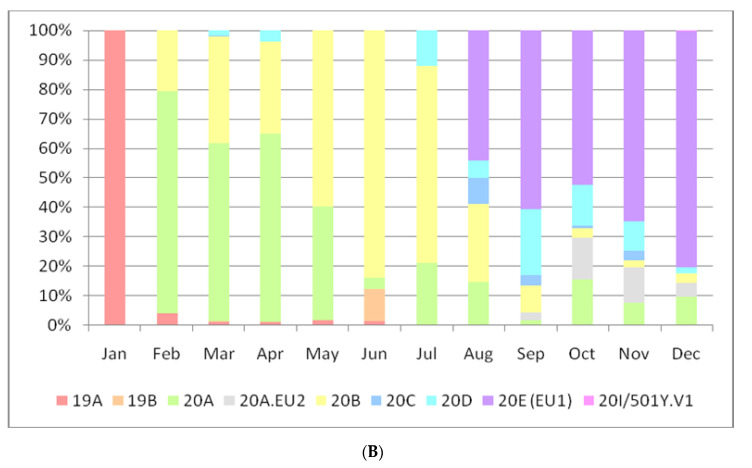
(**A**): Distribution of Nextstrain Clades for 353 SARS-CoV-2 genome sequences collected from the Apulia and Basilicata regions from March 2020 to December 2020. (**B**). Distribution of Nextstrain Clades for 1946 SARS-CoV-2 genome sequences submitted to GISAID from Italy (not including Apulia and Basilicata regions) and collected from January 2020 to December 2020.

To date, a total of 391 SARS-CoV-2 genomes have been sequenced in the Italian regions of Apulia and Basilicata. Among these, we sequenced and submitted 298 genomes, with a reasonable number of mapped reads and level of mean coverage (Supplementary Table S2). Of the 93 remaining genome sequences deposited in GISAID, 55 with <1% Ns were also selected for the analysis. Therefore, we performed our analysis on a total of 353 SARS-CoV-2 selected genome sequences, obtained from samples collected from March 2020 to January 2021. Among these, Nextclade analysis allowed to identify three main clades: 51.27% (*n* = 181) of the genomes belonged to Nextstrain clade 20E(EU1), 29.18% (*n* = 103) belonged to clade 20B, and 9.63% (*n* = 34) belonged to clade 20A. The 5.67% (*n* = 20) of the remaining sequences were assigned to clade 20A.EU2, 2.55% (*n* = 9), to the variant of concern 20I/501Y.V1, and 1.70% (*n* = 6) to clade 20D (Figure 3A). Lineage assignment for all 353 genomes grouped by clades is shown in Supplementary Table S3. These percentages are in accordance with the sequencing data obtained from the other regions of Italy (Figure 3B). It is interesting to highlight that the emerging clade 20E(EU1), whose first Italian genome on repository was detected on 13 August 2020, has exceeded numerically, only in the second wave, the main clades 20A and 20B, circulating in Italy since the beginning of 2020.

Furthermore, phylogeny reported on Nextstrain (https://nextstrain.org/ncov/global, accessed 21 January 2021) showed that, in the global sampling, 20A and 20B (along with the 20C) were the most abundant clades, within the 11 known Nextstrain clades.

Among the 353 genomes collected from Apulia and Basilicata, sequences belonging to more uncommon lineages were detected, also within the most globally widespread clades such as 20A and 20B. The GISAID repository recorded a very low incidence for these lineages, which may represent new emerging variants.

Phylogenetic tree resulting from multiple alignment of all the 2244 SARS-CoV-2 genome sequences submitted to GISAID from Italy and included in this study was obtained and decorated with iTOL [23] (Figure 2). The clustering of genomes evidenced by the phylogenetic tree agrees with the clade assignment. The sequences from Apulia and Basilicata are evenly distributed among those of the other Italian regions, highlighting the import and spread of variants throughout the Country.

## 4. Discussion

Although it is clear that mortality rates are different from region to region and virulence is variable from person to person [24,25,26,27], analysis of the SARS-CoV-2 genome is crucial to understanding the pathogenesis, transmission, and spread of COVID-19. Since the publication of the first SARS-CoV-2 genome, scientists around the world have quickly realized the immediate necessity to obtain larger genetic information from as many viral genomes as possible.

By focusing on the phylogenetic distribution in Italy, we observed that the 20E (EU1) and 20A were the two main clades with frequencies of 31.82% and 30.84%, respectively, immediately followed by clade 20B, with 26.56% of prevalence (Supplementary Table S4, Figure 2).

The SARS-CoV-2 variant 20E(EU1), presumably emerged in Spain and subsequently spread to several European areas, was detected in early summer 2020 [28]. To date, the 18% of genomes detected in Europe between June 2020 and January 2021 belongs to 20E(EU1) clade. In Italy, as of 25 January 2021, in addition to our 181 genomes, other 537 genomes of this clade have been detected. The 86.40% of Italian sequences assigned to this clade, characterized by the A222V amino acid substitution in the Spike protein, belong to the Pangolin B.1.177 lineage, that has been spreading globally in the last months [29]. 

Interestingly, two new emerging clades, 20A.EU1 and 20A.EU2, are gaining relevant prevalence rates in our regions.

Cluster 20A.EU2 emerged during summer 2020 [28] and is characterized by an amino acid substitution S477N on the Spike protein, corresponding to the nucleotide mutation G22992A. All 20 genomes from Apulia and Basilicata associated with clade 20A.EU2 belong to lineage B.1.160, which was detected for the first time in Italy and submitted to GISAID. The number of sequences assigned to lineage B.1.160 has been increasing since the end of August [30].

The last two clades detected in our regions are 20D and 20I/501Y.V1, associated with the one commonly called ‘English variant’. This new rapidly spreading variant, firstly reported in the UK [31,32], originates from the SARS-CoV-2 20B/GR clade (lineage B.1.1.7) and contains multiple mutations, including a combination of the S:N501Y (i.e., an asparagine to tyrosine amino acid substitution at position 501 in the viral S gene) and the S:69–70del (i.e., a deletion of six bases coding for histidine and valine at positions 69 and 70, respectively, in the viral S gene) mutations. The nine samples belonging to this variant, collected from Apulia and Basilicata, were all sampled from Italian patients who returned to these regions from England during the Christmas holidays and from close contacts associated with them.

Among the genome sequenced, we detected several lineages with a low frequency rate based on data available in the GISAID repository.

Three genomes belonging to 20A clade, collected from Brindisi in late October and early November 2020, were assigned to lineage B.1.258.14, of which only 26 other cases were reported worldwide.

Among the 103 genomes belonging to clade 20B, 45 strains were associated with lineage B.1.1.229, first detected in April in Nigeria and of which only 19 other genomes have been reported worldwide, 3 of which were collected in Europe. We sequenced 32 of these 45 genomes classified as B.1.1.229. One of these was isolated from a 10-day-old newborn [33], whereas 23 samples represent a community outbreak: these were 23 cases of infection directly associated with exposure to a single COVID-19 patient during a bus trip. The outbreak that occurred during the bus trip (B.1.1.229 lineage) highlighted the risk of a high rate of SARS-CoV-2 transmission in crowded environments, such as public transport. As already reported [34,35], poor ventilation and draughts generated by air systems may create an ideal setting for such a strong aerosol spread of the new variant of the virus, via respiratory droplets.

The genomic surveillance overview in Apulia and Basilicata regions, during the second peak of infection that began close to the end of summer 2020, has highlighted the emergence of several lineages that were totally absent among the cases collected from January to June 2020 (Figure 1).

Taken together, our results showed a rapid increase in the number of unique viral variants, characterized by different mutations, when compared to the first strain isolated in Wuhan, China, in December 2019, in line with the trend observed in other Countries [36]. Specifically, genomes clustering revealed the emergence of clades 20E(EU1) and 20A.EU2 among SARS-CoV-2 cases sampled in our regions in the South of Italy. We identified and reported for the first time in Italy new emerging lineages, such as B.1.1.115, B.1.1.204, B.1.1.229, B.1.1.277, B.1.1.293, B.1.1.33, and B.1.1.39. Nevertheless, some lineages, as well as the lineage B.1.177, emerged and were detected for the first time in other Countries outside Italy from March to July 2020 (https://cov-lineages.org/lineages, accessed: 21 January 2021). After this period, the previous lineages started to spread in the Italian community. Europe, and especially Italy, are popular summer holidays destinations, and this may have allowed different opportunities for the introduction of new emerging lineages in European Countries. Several studies hypothesize that the spread of the new variants may have been facilitated by the reduction of travel restrictions [37] and border control measures [38]. It is estimated that the weak preventive measures adopted during summer 2020 to limit human-to-human transmission of SARS-CoV-2 have played a crucial role in the global spread of new variants; summer travelers are believed to have carried new clades, such as 20E(EU1), within European Countries [28], probably undermining the local efforts to decrease the number of SARS-CoV-2 cases.

Interestingly, the new lineages detected in Apulia and Basilicata are characterized by non-synonymous mutations in the Spike gene. Some mutations, such as A23403G (D614G), are shared by several clades; others are specific and characteristic of a single clade. These mutations that might affect the structure of the Spike protein are of primary interest [39,40] since many vaccine candidates and serological tests rely on the conformation of this protein. For instance, among our genomes, we detected five sequences of clade 20A belonging to a widespread variant of SARS-CoV-2, characterized by the N439K mutation in the receptor binding motif (RBM) of the SARS-CoV-2 Spike protein. Three of these sequences were collected from Apulia and were associated with lineage B.1.258.14, while the other two samples were collected from Basilicata and belonged to lineage B.1.258. Just three other genomes with this mutation were detected in Italy, collected in Campania. Several studies [41,42] found that the N439K mutation results in immune escape from a panel of neutralizing monoclonal antibodies as well as from polyclonal sera from a sizeable fraction of people recovered from infection.

Similarly, the N501Y mutation of the ‘English variant’ is of great concern because it involves one of the six key amino acid residues determining a tight interaction of the SARS-CoV-2 receptor-binding domain (RBD) with its cellular receptor angiotensin-converting enzyme 2 (ACE2) [43].

These findings might have consequences for the efficacy of emerging vaccines and antibody therapeutics.

It is important to highlight that it is currently unknown which of the characteristic mutations of new variants have an advantage in terms of transmission, viral replication, or reduced immunogenicity, as was widely observed for the D614G substitution, which has over time completely replaced the ancestral wild-type virus [44,45,46]. Indeed, more experimental works and additional genome sequences from Italy and other Countries are required to understand the full spread rate of the new lineages and their putative selective advantages.

To date, in Italy, given the high prevalence of SARS-CoV-2 infection cases, the number of publicly available genomes is very low, so it does not represent the current genetic diversity of the circulating viral population and the distribution of lineages in our geographical area and does not allow predicting the occurrence of new emerging clades [47].

According to these findings, it is clear that the improvement of genomic surveillance is fundamental to understand the spread of SARS-CoV-2 in different regions, to rapidly identify potential global transmission networks, and to consolidate response strategies. It is only through sequencing the viral genome that we can detect new SARS-CoV-2 variants and monitor their spread within and between Countries. The updating of genome sequences in real time allows tracking the most recent genetic evolution of the virus and the diffusion of emerging clades. The number of available sequences varies widely between Countries, and we might be able to identify emerging variants sooner with faster and more regular sequencing efforts across Europe and the world.

## Data Availability

Data available in a publicly accessible repository that does not release DOIs: The datasets obtained in this study have been made publicly available. These data can be found on the official GISAID repository, here: [https://www.gisaid.org/] (accessed on 20 April 2021). The Accession ID of the sequences obtained in this study can be found in Supplementary Table S1.

## Supplementary Materials

The following are available online at https://www.mdpi.com/article/10.3390/v13050731/s1, Table S1: Data of 298 human SARS-CoV-2 genomes collected from Apulia and Basilicata, sequenced in this study and submitted to GISAID (Accession ID); Table S2: Quality (raw sequencing) data for the 144 SARS-CoV-2 genomes sequenced in this study; Table S3: Pangolin lineage prediction for the 353 SARS-CoV-2 genomic sequences from Apulia and Basilicata, surveyed in this study. The percentages refer to the frequency compared to the total number of genomes assigned to the same clade; Table S4: Nextclade prediction for the 2244 Italian sequences of the SARS-CoV-2 genome and the 353 sequences from Apulia and Basilicata, with the corresponding incidence rates. Supplementary File 2: A newick (plain text) version of the phylogenetic tree, with branch lengths and support values at nodes.
