# Supplementary material for: Genomic Surveillance of Circulating SARS-CoV-2 in South East Italy: A One-Year Retrospective Genetic Study
Source: Viruses. 2021 Apr 22;13(5):731. doi: 10.3390/v13050731 (PMC8147059; doi:10.3390/v13050731)
Supplement: Supplementary file 1 [file viruses-13-00731-s001.zip › viruses-1160838-Supplementary File 1.pdf]

viruses

MDPI

Supplementary Materials: The following are available online at www.mdpi.com /xxx, **Table S1**: Data of 298 human SARS-CoV-2 genomes collected from Apulia and Basilicata, sequenced in this study and submitted to GISAID (Accession ID); **Table S2**: Quality (raw sequencing) data for the 144 SARS-CoV-2 genomes sequenced in this study; **Table S3**: Pangolin lineage prediction for the 353 SARS-CoV-2 genomic sequences from Apulia and Basilicata, surveyed in this study. The percentages refer to the frequency compared to the total number of genomes assigned to the same clade; **Table S4:** Nextclade prediction for the 2244 Italian sequences of the SARS-CoV-2 genome and the 353 sequences from Apulia and Basilicata, with the corresponding incidence rates.

Virus name	Accession ID	Collection date	Location	Lineage
hCoV-19/Italy/APU-IZSPB-59PT/2020	EPI_ISL_876811	21/04/2020	Europe / Italy / Apulia / Bari / Adelfia	B.1.1.74
hCoV-19/Italy/APU-IZSPB-60PT/2020	EPI_ISL_876812	21/04/2020	Europe / Italy / Apulia / Bari / Bitetto	B.1.1.74
hCoV-19/Italy/APU-IZSPB-67PT/2020	EPI_ISL_876739	11/04/2020	Europe / Italy / Apulia / Brindisi	B.1.1.265
hCoV-19/Italy/APU-IZSPB-74PT/2020	EPI_ISL_876813	11/04/2020	Europe / Italy / Apulia / Brindisi	B.1.1.74
hCoV-19/Italy/APU-IZSPB-150PT/2020	EPI_ISL_722899	2020	Europe / Italy / Apulia / Lecce / Copertino	B.1.1.33
hCoV-19/Italy/APU-IZSPB-158PT/2020	EPI_ISL_722872	18/07/2020	Europe / Italy / Apulia / Brindisi / Fasano	B.1.1.305
hCoV-19/Italy/APU-IZSPB_171PT/2020	EPI_ISL_653823	17/08/2020	Europe / Italy / Apulia / Foggia / Manfredonia	B.1.1.293
hCoV-19/Italy/APU-IZSPB-178PT/2020	EPI_ISL_722859	28/08/2020	Europe / Italy / Apulia / Bari	B.1.1.229
hCoV-19/Italy/APU-IZSPB-180PT/2020	EPI_ISL_722898	28/08/2020	Europe / Italy / Apulia / Bari	B.1.1.229
hCoV-19/Italy/APU-IZSPB-181PT/2020	EPI_ISL_722860	28/08/2020	Europe / Italy / Apulia / Bari	B.1.1.229
hCoV-19/Italy/APU-IZSPB-182PT/2020	EPI_ISL_649785	28/08/2020	Europe / Italy / Apulia	B.1.1.229
hCoV-19/Italy/APU-IZSPB-183PT/2020	EPI_ISL_722861	28/08/2020	Europe / Italy / Apulia / Bari / Torre a Mare	B.1.1.229
hCoV-19/Italy/APU-IZSPB-184PT/2020	EPI_ISL_722862	28/08/2020	Europe / Italy / Apulia / Bari	B.1.1.229
hCoV-19/Italy/APU-IZSPB-185PT/2020	EPI_ISL_722863	28/08/2020	Europe / Italy / Apulia / Bari	B.1.1.229
hCoV-19/Italy/APU-IZSPB-186PT/2020	EPI_ISL_722864	28/08/2020	Europe / Italy / Apulia / Bari	B.1.1.288
hCoV-19/Italy/APU-IZSPB-187PT/2020	EPI_ISL_722895	28/08/2020	Europe / Italy / Apulia / Bari	B.1.1.229
hCoV-19/Italy/APU-IZSPB-188PT/2020	EPI_ISL_722865	28/08/2020	Europe / Italy / Apulia / Bari	B.1.1.229
hCoV-19/Italy/APU-IZSPB-189PT/2020	EPI_ISL_722866	28/08/2020	Europe / Italy / Apulia / Bari	B.1.1.229
hCoV-19/Italy/APU-IZSPB-190PT/2020	EPI_ISL_649938	28/08/2020	Europe / Italy / Apulia / Bari	B.1.1.229
hCoV-19/Italy/APU-IZSPB-192PT/2020	EPI_ISL_649939	28/08/2020	Europe / Italy / Apulia / Bari	B.1.1.229
hCoV-19/Italy/BAS-IZSPB-194PT/2020	EPI_ISL_722892	24/08/2020	Europe / Italy / Basilicata / Potenza	B.1
hCoV-19/Italy/APU-IZSPB-195PT/2020	EPI_ISL_722867	28/08/2020	Europe / Italy / Apulia / Bari	B.1.1.229
hCoV-19/Italy/APU-IZSPB-196PT/2020	EPI_ISL_722900	28/08/2020	Europe / Italy / Apulia / Bari	B.1.1.229
hCoV-19/Italy/APU-IZSPB-197PT/2020	EPI_ISL_722868	28/08/2020	Europe / Italy / Apulia / Bari	B.1.1.229
hCoV-19/Italy/APU-IZSPB_198PT/2020	EPI_ISL_653763	28/08/2020	Europe / Italy / Apulia / Bari	B.1.1.229
hCoV-19/Italy/APU-IZSPB_200PT/2020	EPI_ISL_653764	28/08/2020	Europe / Italy / Apulia / Bari	B.1.1.229
hCoV-19/Italy/APU-IZSPB-201PT/2020	EPI_ISL_722869	28/08/2020	Europe / Italy / Apulia / Bari	B.1.1.229
hCoV-19/Italy/APU-IZSPB-202PT/2020	EPI_ISL_722870	28/08/2020	Europe / Italy / Apulia / Bari	B.1.1.229
hCoV-19/Italy/APU-IZSPB-203PT/2020	EPI_ISL_722871	28/08/2020	Europe / Italy / Apulia / Bari	B.1.1.1
hCoV-19/Italy/APU-IZSPB_265PT/2020	EPI_ISL_653784	22/09/2020	Europe / Italy / Apulia / Bari	B.1.1.277
hCoV-19/Italy/APU-IZSPB_266PT/2020	EPI_ISL_653785	29/09/2020	Europe / Italy / Apulia / Foggia	B.1.1.277
hCoV-19/Italy/APU-IZSPB_267PT/2020	EPI_ISL_653786	30/09/2020	Europe / Italy / Apulia / Bari / Gravina di Puglia	B.1.177
hCoV-19/Italy/APU-IZSPB_269PT/2020	EPI_ISL_653765	02/11/2020	Europe / Italy / Apulia / Bari / Acquaviva delle Fonti	B.1.177
hCoV-19/Italy/APU-IZSPB_271PT/2020	EPI_ISL_653766	02/11/2020	Europe / Italy / Apulia / Brindisi	B.1.160
hCoV-19/Italy/APU-IZSPB_272PT/2020	EPI_ISL_653767	02/11/2020	Europe / Italy / Apulia / Bari / Castellana Grotte	B.1.160
hCoV-19/Italy/APU-IZSPB_274PT/2020	EPI_ISL_653768	02/11/2020	Europe / Italy / Apulia / Brindisi / Ostuni	B.1.177
hCoV-19/Italy/APU-IZSPB_275PT/2020	EPI_ISL_653769	02/11/2020	Europe / Italy / Apulia / Brindisi / Francavilla Fontana	B.1.177
hCoV-19/Italy/APU-IZSPB_276PT/2020	EPI_ISL_653770	02/11/2020	Europe / Italy / Apulia / Brindisi	B.1.160
hCoV-19/Italy/APU-IZSPB_277PT/2020	EPI_ISL_653771	02/11/2020	Europe / Italy / Apulia / Brindisi	B.1.160
hCoV-19/Italy/APU-IZSPB_278PT/2020	EPI_ISL_653772	02/11/2020	Europe / Italy / Apulia / Brindisi	B.1.160
hCoV-19/Italy/APU-IZSPB_280PT/2020	EPI_ISL_653773	02/11/2020	Europe / Italy / Apulia / Brindisi / Francavilla Fontana	B.1.177
hCoV-19/Italy/APU-IZSPB_281PT/2020	EPI_ISL_653774	02/11/2020	Europe / Italy / Apulia / Brindisi / Francavilla Fontana	B.1.177
hCoV-19/Italy/APU-IZSPB_282PT/2020	EPI_ISL_653775	02/11/2020	Europe / Italy / Apulia / Brindisi	B.1.177
hCoV-19/Italy/APU-IZSPB_284PT/2020	EPI_ISL_653776	02/11/2020	Europe / Italy / Apulia / Brindisi	B.1.160
hCoV-19/Italy/APU-IZSPB_285PT/2020	EPI_ISL_653777	02/11/2020	Europe / Italy / Apulia / Brindisi / San Pietro Vernotico	B.1.160
hCoV-19/Italy/APU-IZSPB_286PT/2020	EPI_ISL_653778	02/11/2020	Europe / Italy / Apulia / Brindisi / San Pietro Vernotico	B.1.160
hCoV-19/Italy/APU-IZSPB_287PT/2020	EPI_ISL_653779	02/11/2020	Europe / Italy / Apulia / Brindisi / Erchia	B.1.177
hCoV-19/Italy/APU-IZSPB_289PT/2020	EPI_ISL_653780	02/11/2020	Europe / Italy / Apulia / Brindisi	B.1.258.14
hCoV-19/Italy/APU-IZSPB_290PT/2020	EPI_ISL_653781	02/11/2020	Europe / Italy / Apulia / Brindisi / Ostuni	B.1.177
hCoV-19/Italy/APU-IZSPB_291PT/2020	EPI_ISL_653782	30/10/2020	Europe / Italy / Apulia / Brindisi / Fasano	B.1.1.1

hCoV-19/Italy/APU-IZSPB_292PT/2020	EPI_ISL_653783	02/11/2020	Europe / Italy / Apulia / Brindisi	B.1.160
hCoV-19/Italy/APU-IZSPB_293PT/2020	EPI_ISL_653787	03/11/2020	Europe / Italy / Apulia / Taranto / Martina Franca	B.1.1.1
hCoV-19/Italy/APU-IZSPB_294PT/2020	EPI_ISL_653788	03/11/2020	Europe / Italy / Apulia / Bari / Monopoli	B.1.177
hCoV-19/Italy/APU-IZSPB_295PT/2020	EPI_ISL_653789	03/11/2020	Europe / Italy / Apulia / Martina Franca	B.1.1.1
hCoV-19/Italy/APU-IZSPB_296PT/2020	EPI_ISL_653790	03/11/2020	Europe / Italy / Apulia / Martina Franca	B.1.1.1
hCoV-19/Italy/APU-IZSPB_297PT/2020	EPI_ISL_653791	03/11/2020	Europe / Italy / Apulia / Bari / Altamura	B.1.1.229
hCoV-19/Italy/BAS-IZSPB_298PT/2020	EPI_ISL_653792	03/11/2020	Europe / Italy / Basilicata / Matera / Ferrandina	B.1.1.277
hCoV-19/Italy/APU-IZSPB_299PT/2020	EPI_ISL_653793	03/11/2020	Europe / Italy / Apulia / Bari	B.1.160
hCoV-19/Italy/APU-IZSPB_300PT/2020	EPI_ISL_653794	03/11/2020	Europe / Italy / Apulia / Bari / Turi	B.1.1.277
hCoV-19/Italy/APU-IZSPB_301PT/2020	EPI_ISL_653795	03/11/2020	Europe / Italy / Apulia / Bari / Monopoli	B.1.416
hCoV-19/Italy/APU-IZSPB_302PT/2020	EPI_ISL_653796	03/11/2020	Europe / Italy / Apulia / Bari / Acquaviva delle Fonti	B.1.177
hCoV-19/Italy/APU-IZSPB_303PT/2020	EPI_ISL_653797	03/11/2020	Europe / Italy / Apulia / Bari / Acquaviva delle Fonti	B.1.177
hCoV-19/Italy/APU-IZSPB_304PT/2020	EPI_ISL_653798	03/11/2020	Europe / Italy / Apulia / Bari / Acquaviva delle Fonti	B.1.177
hCoV-19/Italy/APU-IZSPB_305PT/2020	EPI_ISL_653799	03/11/2020	Europe / Italy / Apulia / Bari / Locorotondo	B.1.177
hCoV-19/Italy/APU-IZSPB_306PT/2020	EPI_ISL_653800	03/11/2020	Europe / Italy / Apulia / Bari / Alberobello	B.1.1.39
hCoV-19/Italy/APU-IZSPB_307PT/2020	EPI_ISL_653801	02/11/2020	Europe / Italy / Apulia / Brindisi / Latiano	B.1.177
hCoV-19/Italy/APU-IZSPB_308PT/2020	EPI_ISL_653802	24/10/2020	Europe / Italy / Apulia / Bari / Monopoli	B.1.1.229
hCoV-19/Italy/APU-IZSPB_309PT/2020	EPI_ISL_653803	24/10/2020	Europe / Italy / Apulia / Bari / Monopoli	B.1.1.229
hCoV-19/Italy/APU-IZSPB_310PT/2020	EPI_ISL_653804	03/11/2020	Europe / Italy / Apulia / Bari / Sammichele di Bari	B.1.177
hCoV-19/Italy/APU-IZSPB_311PT/2020	EPI_ISL_653805	03/11/2020	Europe / Italy / Apulia / Bari / Monopoli	B.1.1.229
hCoV-19/Italy/APU-IZSPB_312PT/2020	EPI_ISL_653806	03/11/2020	Europe / Italy / Apulia / Bari / Putignano	B.1.177
hCoV-19/Italy/APU-IZSPB_313PT/2020	EPI_ISL_653807	02/11/2020	Europe / Italy / Apulia / Brindisi / Ceglie Messapica	B.1.160
hCoV-19/Italy/APU-IZSPB_314PT/2020	EPI_ISL_653808	02/11/2020	Europe / Italy / Apulia / Brindisi / Carovigno	B.1.177
hCoV-19/Italy/APU-IZSPB_315PT/2020	EPI_ISL_653809	02/11/2020	Europe / Italy / Apulia / Brindisi	B.1.160
hCoV-19/Italy/APU-IZSPB_316PT/2020	EPI_ISL_653810	31/10/2020	Europe / Italy / Apulia / Brindisi	B.1.258.14
hCoV-19/Italy/APU-IZSPB_317PT/2020	EPI_ISL_653811	02/11/2020	Europe / Italy / Apulia / Brindisi	B.1.258.14
hCoV-19/Italy/APU-IZSPB_318PT/2020	EPI_ISL_653812	02/11/2020	Europe / Italy / Apulia / Brindisi / Fasano	B.1.177
hCoV-19/Italy/APU-IZSPB_319PT/2020	EPI_ISL_653813	11/04/2020	Europe / Italy / Apulia / Brindisi	B.1.1.74
hCoV-19/Italy/APU-IZSPB_321PT/2020	EPI_ISL_653814	11/04/2020	Europe / Italy / Apulia / Brindisi	B.1.1.277
hCoV-19/Italy/APU-IZSPB_322PT/2020	EPI_ISL_653815	11/04/2020	Europe / Italy / Apulia / Brindisi	B.1.1.74
hCoV-19/Italy/APU-IZSPB_323PT/2020	EPI_ISL_653816	11/04/2020	Europe / Italy / Apulia / Brindisi	B.1.1.74
hCoV-19/Italy/APU-IZSPB_324PT/2020	EPI_ISL_653817	11/04/2020	Europe / Italy / Apulia / Brindisi	B.1.1.74
hCoV-19/Italy/APU-IZSPB_325PT/2020	EPI_ISL_653818	11/04/2020	Europe / Italy / Apulia / Brindisi	B.1.1.74
hCoV-19/Italy/APU-IZSPB_326PT/2020	EPI_ISL_653819	04/11/2020	Europe / Italy / Apulia / Bari	B.1.177
hCoV-19/Italy/APU-IZSPB_327PT/2020	EPI_ISL_653820	06/11/2020	Europe / Italy / Apulia / Bari / Putignano	B.1.160
hCoV-19/Italy/APU-IZSPB_328PT/2020	EPI_ISL_653821	27/10/2020	Europe / Italy / Apulia / Bari	B.1.1.277
hCoV-19/Italy/APU-IZSPB_329PT/2020	EPI_ISL_653822	04/11/2020	Europe / Italy / Apulia / Bari	B.1.177
hCoV-19/Italy/APU-IZSPB-330PT/2020	EPI_ISL_722908	21/10/2020	Europe / Italy / Apulia / Foggia	B.1.177
hCoV-19/Italy/APU-IZSPB-331PT/2020	EPI_ISL_722911	05/11/2020	Europe / Italy / Apulia / Foggia / Lucera	B.1.177
hCoV-19/Italy/APU-IZSPB-332PT/2020	EPI_ISL_722893	10/10/2020	Europe / Italy / Apulia / Foggia	B.1.416
hCoV-19/Italy/APU-IZSPB-333PT/2020	EPI_ISL_722894	05/11/2020	Europe / Italy / Apulia / Foggia / Lucera	B.1.177
hCoV-19/Italy/APU-IZSPB-334PT/2020	EPI_ISL_722909	04/11/2020	Europe / Italy / Apulia / Foggia / Lucera	B.1.177
hCoV-19/Italy/APU-IZSPB-335PT/2020	EPI_ISL_722910	20/11/2020	Europe / Italy / Apulia / Foggia	B.1.160
hCoV-19/Italy/BAS-IZSPB-336PT/2020	EPI_ISL_722901	28/10/2020	Europe / Italy / Basilicata / Matera / Montalbano Jonico	B.1.177
hCoV-19/Italy/BAS-IZSPB-338PT/2020	EPI_ISL_722873	27/10/2020	Europe / Italy / Basilicata / Matera / Policoro	B.1.258
hCoV-19/Italy/BAS-IZSPB-339PT/2020	EPI_ISL_722874	29/10/2020	Europe / Italy / Basilicata / Matera / Garaguso	B.1.1.229
hCoV-19/Italy/BAS-IZSPB-341PT/2020	EPI_ISL_722875	28/10/2020	Europe / Italy / Basilicata / Matera	B.1.177
hCoV-19/Italy/BAS-IZSPB-351PT/2020	EPI_ISL_722876	28/10/2020	Europe / Italy / Basilicata / Matera	B.1.177
hCoV-19/Italy/BAS-IZSPB-352PT/2020	EPI_ISL_722913	28/10/2020	Europe / Italy / Basilicata / Matera	B.1.177
hCoV-19/Italy/BAS-IZSPB-356PT/2020	EPI_ISL_722914	28/10/2020	Europe / Italy / Basilicata / Matera	B.1.160
hCoV-19/Italy/BAS-IZSPB-357PT/2020	EPI_ISL_722902	27/10/2020	Europe / Italy / Basilicata / Matera / Policoro	B.1.258
hCoV-19/Italy/BAS-IZSPB-358PT/2020	EPI_ISL_722903	29/10/2020	Europe / Italy / Basilicata / Potenza / Lauria	B.1.1.229
hCoV-19/Italy/BAS-IZSPB-359PT/2020	EPI_ISL_722877	29/10/2020	Europe / Italy / Basilicata / Potenza	B.1.160
hCoV-19/Italy/BAS-IZSPB-364PT/2020	EPI_ISL_722878	15/10/2020	Europe / Italy / Basilicata / Matera / Calciano	B.1.177
hCoV-19/Italy/BAS-IZSPB-365PT/2020	EPI_ISL_722915	15/10/2020	Europe / Italy / Basilicata / Matera / Tricarico	B.1.177
hCoV-19/Italy/BAS-IZSPB-366PT/2020	EPI_ISL_722916	15/10/2020	Europe / Italy / Basilicata / Matera / Calciano	B.1.177
hCoV-19/Italy/BAS-IZSPB-368PT/2020	EPI_ISL_722879	16/10/2020	Europe / Italy / Basilicata / Potenza / Noepoli	B.1.177
hCoV-19/Italy/BAS-IZSPB-370PT/2020	EPI_ISL_722904	16/10/2020	Europe / Italy / Basilicata / Potenza / Roccanova	B.1.177
hCoV-19/Italy/BAS-IZSPB-371PT/2020	EPI_ISL_722917	16/10/2020	Europe / Italy / Basilicata / Matera	B.1.177

hCoV-19/Italy/BAS-IZSPB-372PT/2020	EPI_ISL_722905	28/10/2020	Europe / Italy / Basilicata / Matera / Irsina	B.1.177
hCoV-19/Italy/BAS-IZSPB-373PT/2020	EPI_ISL_722880	16/10/2020	Europe / Italy / Basilicata / Matera / Miglionico	B.1.177
hCoV-19/Italy/BAS-IZSPB-374PT/2020	EPI_ISL_722881	16/10/2020	Europe / Italy / Basilicata / Matera / Miglionico	B.1.177
hCoV-19/Italy/APU-IZSPB-377PT/2020	EPI_ISL_722918	14/10/2020	Europe / Italy / Apulia / Bari	B.1.177
hCoV-19/Italy/BAS-IZSPB-378PT/2020	EPI_ISL_722919	17/10/2020	Europe / Italy / Basilicata / Matera	B.1.177
hCoV-19/Italy/BAS-IZSPB-379PT/2020	EPI_ISL_722906	26/10/2020	Europe / Italy / Basilicata / Matera / Accettura	B.1.1.229
hCoV-19/Italy/BAS-IZSPB-380PT/2020	EPI_ISL_722882	25/10/2020	Europe / Italy / Basilicata / Matera	B.1.177
hCoV-19/Italy/BAS-IZSPB-381PT/2020	EPI_ISL_722883	26/10/2020	Europe / Italy / Basilicata / Matera	B.1.177
hCoV-19/Italy/BAS-IZSPB-382PT/2020	EPI_ISL_722907	19/10/2020	Europe / Italy / Basilicata / Matera	B.1.177
hCoV-19/Italy/BAS-IZSPB-383PT/2020	EPI_ISL_722884	25/10/2020	Europe / Italy / Basilicata / Matera	B.1.177
hCoV-19/Italy/BAS-IZSPB-384PT/2020	EPI_ISL_722885	20/10/2020	Europe / Italy / Basilicata / Matera / Rotondella	B.1.177
hCoV-19/Italy/BAS-IZSPB-385PT/2020	EPI_ISL_722886	20/10/2020	Europe / Italy / Basilicata / Potenza / Roccanova	B.1.177
hCoV-19/Italy/BAS-IZSPB-386PT/2020	EPI_ISL_722920	25/10/2020	Europe / Italy / Basilicata / Matera	B.1.177
hCoV-19/Italy/BAS-IZSPB-387PT/2020	EPI_ISL_722921	20/10/2020	Europe / Italy / Basilicata / Matera / Irsina	B.1.177
hCoV-19/Italy/BAS-IZSPB-388PT/2020	EPI_ISL_722887	26/10/2020	Europe / Italy / Basilicata / Matera	B.1.177
hCoV-19/Italy/BAS-IZSPB-389PT/2020	EPI_ISL_722888	25/10/2020	Europe / Italy / Basilicata / Potenza / Roccanova	B.1.177
hCoV-19/Italy/BAS-IZSPB-390PT/2020	EPI_ISL_722889	25/10/2020	Europe / Italy / Basilicata / Matera	B.1.177
hCoV-19/Italy/BAS-IZSPB-391PT/2020	EPI_ISL_722890	25/10/2020	Europe / Italy / Basilicata / Matera	B.1.177
hCoV-19/Italy/BAS-IZSPB-392PT/2020	EPI_ISL_722922	24/10/2020	Europe / Italy / Basilicata / Matera / Tursi	B.1.177
hCoV-19/Italy/BAS-IZSPB-393PT/2020	EPI_ISL_722923	25/10/2020	Europe / Italy / Basilicata / Potenza / Roccanova	B.1.177
hCoV-19/Italy/BAS-IZSPB-395PT/2020	EPI_ISL_722924	21/10/2020	Europe / Italy / Basilicata / Matera / Irsina	B.1.177
hCoV-19/Italy/BAS-IZSPB-397PT/2020	EPI_ISL_722925	21/10/2020	Europe / Italy / Basilicata / Matera / Irsina	B.1.177
hCoV-19/Italy/BAS-IZSPB-398PT/2020	EPI_ISL_722891	25/10/2020	Europe / Italy / Basilicata / Matera / Montescaglioso	B.1.177
hCoV-19/Italy/APU-IZSPB-399PT/2020	EPI_ISL_745193	21/12/2020	Europe / Italy / Apulia / Bari / Casamassima	B.1.1.7
hCoV-19/Italy/APU-IZSPB-400PT/2020	EPI_ISL_745192	21/12/2020	Europe / Italy / Apulia / Bari / Molfetta	B.1.1.7
hCoV-19/Italy/APU-IZSPB_404PT/2020	EPI_ISL_794746	24/12/2020	Europe / Italy / Apulia / Bari	B.1.177
hCoV-19/Italy/APU-IZSPB_405PT/2020	EPI_ISL_794747	23/12/2020	Europe / Italy / Apulia / Lecce	B.1.1.7
hCoV-19/Italy/APU-IZSPB_406PT/2020	EPI_ISL_794748	24/12/2020	Europe / Italy / Apulia / Bari	B.1.1.7
hCoV-19/Italy/APU-IZSPB_407PT/2020	EPI_ISL_794749	25/11/2020	Europe / Italy / Apulia / Bari	B.1
hCoV-19/Italy/APU-IZSPB_408PT/2020	EPI_ISL_794750	22/12/2020	Europe / Italy / Apulia / Lecce	B.1.1.7
hCoV-19/Italy/APU-IZSPB_409PT/2020	EPI_ISL_794751	23/12/2020	Europe / Italy / Apulia / Lecce	B.1.1.7
hCoV-19/Italy/APU-IZSPB_411PT/2020	EPI_ISL_794752	23/12/2020	Europe / Italy / Apulia / BAT	B.1.1.204
hCoV-19/Italy/APU-IZSPB_412PT/2020	EPI_ISL_794753	22/12/2020	Europe / Italy / Apulia / Lecce	B.1.177
hCoV-19/Italy/APU-IZSPB_413PT/2020	EPI_ISL_794754	16/12/2020	Europe / Italy / Apulia / Foggia	B.1.177
hCoV-19/Italy/APU-IZSPB_414PT/2020	EPI_ISL_794755	25/11/2020	Europe / Italy / Apulia / Foggia	B.1
hCoV-19/Italy/APU-IZSPB_415PT/2020	EPI_ISL_794756	22/12/2020	Europe / Italy / Apulia / Brindisi	B.1.160
hCoV-19/Italy/APU-IZSPB_417PT/2020	EPI_ISL_794757	22/12/2020	Europe / Italy / Apulia / Brindisi	B.1.160
hCoV-19/Italy/APU-IZSPB_418PT/2020	EPI_ISL_794758	23/12/2020	Europe / Italy / Apulia / Brindisi	B.1.177
hCoV-19/Italy/APU-IZSPB-421PT/2020	EPI_ISL_876738	29/12/2020	Europe / Italy / Apulia / Taranto / Martina Franca	B.1.177
hCoV-19/Italy/APU-IZSPB_423PT/2020	EPI_ISL_794759	22/12/2020	Europe / Italy / Apulia / Foggia	B.1.177
hCoV-19/Italy/APU-IZSPB_424PT/2020	EPI_ISL_794760	11/12/2020	Europe / Italy / Apulia / Foggia	B.1.177
hCoV-19/Italy/APU-IZSPB_425PT/2020	EPI_ISL_794761	11/12/2020	Europe / Italy / Apulia / Foggia	B.1.177
hCoV-19/Italy/APU-IZSPB_426PT/2020	EPI_ISL_794762	11/12/2020	Europe / Italy / Apulia / Foggia	B.1.177
hCoV-19/Italy/APU-IZSPB-427PT/2020	EPI_ISL_876800	11/12/2020	Europe / Italy / Apulia / Foggia	B.1.177
hCoV-19/Italy/APU-IZSPB-428PT/2020	EPI_ISL_876801	11/12/2020	Europe / Italy / Apulia / Foggia	B.1.177
hCoV-19/Italy/APU-IZSPB-429PT/2020	EPI_ISL_876802	11/12/2020	Europe / Italy / Apulia / Foggia	B.1.177
hCoV-19/Italy/APU-IZSPB-430PT/2020	EPI_ISL_876803	11/12/2020	Europe / Italy / Apulia / Foggia	B.1.177
hCoV-19/Italy/APU-IZSPB_431PT/2020	EPI_ISL_794763	11/12/2020	Europe / Italy / Apulia / Foggia	B.1.177
hCoV-19/Italy/APU-IZSPB_432PT/2020	EPI_ISL_794764	11/12/2020	Europe / Italy / Apulia / Foggia	B.1.177.8
hCoV-19/Italy/APU-IZSPB_433PT/2020	EPI_ISL_794765	11/12/2020	Europe / Italy / Apulia / Foggia	B.1.177
hCoV-19/Italy/APU-IZSPB_434PT/2020	EPI_ISL_794766	11/12/2020	Europe / Italy / Apulia / Foggia	B.1.177
hCoV-19/Italy/APU-IZSPB_435PT/2020	EPI_ISL_794767	11/12/2020	Europe / Italy / Apulia / Foggia	B.1.177
hCoV-19/Italy/APU-IZSPB_436PT/2020	EPI_ISL_794768	11/12/2020	Europe / Italy / Apulia / Foggia	B.1.177
hCoV-19/Italy/APU-IZSPB_437PT/2020	EPI_ISL_794769	11/12/2020	Europe / Italy / Apulia / Foggia	B.1.177
hCoV-19/Italy/APU-IZSPB_438PT/2020	EPI_ISL_794770	11/12/2020	Europe / Italy / Apulia / Foggia	B.1.177
hCoV-19/Italy/APU-IZSPB-439PT/2020	EPI_ISL_876804	11/12/2020	Europe / Italy / Apulia / Foggia	B.1.177
hCoV-19/Italy/APU-IZSPB-440PT/2020	EPI_ISL_876805	11/12/2020	Europe / Italy / Apulia / Foggia	B.1.177
hCoV-19/Italy/APU-IZSPB-441PT/2020	EPI_ISL_876806	11/12/2020	Europe / Italy / Apulia / Foggia	B.1.177
hCoV-19/Italy/APU-IZSPB-442PT/2020	EPI_ISL_876807	11/12/2020	Europe / Italy / Apulia / Foggia	B.1.177

hCoV-19/Italy/APU-IZSPB_443PT/2020	EPI_ISL_794771	12/12/2020	Europe / Italy / Apulia / Foggia	B.1.177
hCoV-19/Italy/APU-IZSPB_444PT/2020	EPI_ISL_794772	12/12/2020	Europe / Italy / Apulia / Foggia	B.1.177
hCoV-19/Italy/APU-IZSPB_445PT/2020	EPI_ISL_794773	12/12/2020	Europe / Italy / Apulia / Foggia	B.1.177
hCoV-19/Italy/APU-IZSPB_447PT/2020	EPI_ISL_794774	17/12/2020	Europe / Italy / Apulia / Foggia	B.1.177
hCoV-19/Italy/APU-IZSPB_448PT/2020	EPI_ISL_794775	17/12/2020	Europe / Italy / Apulia / Foggia	B.1.177
hCoV-19/Italy/APU-IZSPB_449PT/2020	EPI_ISL_794776	17/12/2020	Europe / Italy / Apulia / Foggia	B.1.177
hCoV-19/Italy/APU-IZSPB_450PT/2020	EPI_ISL_794777	16/12/2020	Europe / Italy / Apulia / Foggia	B.1.177
hCoV-19/Italy/APU-IZSPB_451PT/2020	EPI_ISL_794778	17/12/2020	Europe / Italy / Apulia / Foggia	B.1.1.39
hCoV-19/Italy/APU-IZSPB_452PT/2020	EPI_ISL_794779	22/12/2020	Europe / Italy / Apulia / Foggia	B.1.416
hCoV-19/Italy/APU-IZSPB_453PT/2020	EPI_ISL_794780	23/12/2020	Europe / Italy / Apulia / Foggia	B.1.177
hCoV-19/Italy/APU-IZSPB_454PT/2020	EPI_ISL_794781	23/12/2020	Europe / Italy / Apulia / Foggia	B.1.177
hCoV-19/Italy/APU-IZSPB_455PT/2020	EPI_ISL_794782	23/12/2020	Europe / Italy / Apulia / Foggia	B.1.177
hCoV-19/Italy/APU-IZSPB_456PT/2020	EPI_ISL_794783	23/12/2020	Europe / Italy / Apulia / Foggia	B.1.177
hCoV-19/Italy/APU-IZSPB_457PT/2020	EPI_ISL_794784	17/12/2020	Europe / Italy / Apulia / Foggia	B.1.177
hCoV-19/Italy/APU-IZSPB_458PT/2020	EPI_ISL_794785	23/12/2020	Europe / Italy / Apulia / Foggia	B.1.177
hCoV-19/Italy/APU-IZSPB_459PT/2020	EPI_ISL_794786	23/12/2020	Europe / Italy / Apulia / Foggia	B.1.177
hCoV-19/Italy/APU-IZSPB_460PT/2020	EPI_ISL_794787	23/12/2020	Europe / Italy / Apulia / Foggia	B.1.177
hCoV-19/Italy/APU-IZSPB_461PT/2020	EPI_ISL_794788	14/12/2020	Europe / Italy / Apulia / Foggia	B.1.160
hCoV-19/Italy/APU-IZSPB_462PT/2020	EPI_ISL_794789	15/12/2020	Europe / Italy / Apulia / Foggia	B.1.177
hCoV-19/Italy/APU-IZSPB_463PT/2020	EPI_ISL_794790	14/12/2020	Europe / Italy / Apulia / Foggia	B.1.416
hCoV-19/Italy/APU-IZSPB_464PT/2020	EPI_ISL_794791	12/12/2020	Europe / Italy / Apulia / Foggia	B.1.177
hCoV-19/Italy/APU-IZSPB_465PT/2020	EPI_ISL_794792	12/12/2020	Europe / Italy / Apulia / Foggia	B.1.177
hCoV-19/Italy/APU-IZSPB_466PT/2020	EPI_ISL_794793	14/12/2020	Europe / Italy / Apulia / Foggia	B.1.177
hCoV-19/Italy/APU-IZSPB_467PT/2020	EPI_ISL_794794	17/12/2020	Europe / Italy / Apulia / Foggia	B.1.177
hCoV-19/Italy/APU-IZSPB_468PT/2020	EPI_ISL_794795	17/12/2020	Europe / Italy / Apulia / Foggia	B.1.177
hCoV-19/Italy/APU-IZSPB_469PT/2020	EPI_ISL_794796	17/12/2020	Europe / Italy / Apulia / Foggia	B.1.177
hCoV-19/Italy/APU-IZSPB_470PT/2020	EPI_ISL_794797	17/12/2020	Europe / Italy / Apulia / Foggia	B.1.177
hCoV-19/Italy/APU-IZSPB_471PT/2020	EPI_ISL_794798	17/12/2020	Europe / Italy / Apulia / Foggia	B.1.177
hCoV-19/Italy/APU-IZSPB_472PT/2020	EPI_ISL_794799	17/12/2020	Europe / Italy / Apulia / Foggia	B.1.416
hCoV-19/Italy/APU-IZSPB_473PT/2020	EPI_ISL_794800	16/12/2020	Europe / Italy / Apulia / Foggia	B.1.177
hCoV-19/Italy/APU-IZSPB_474PT/2020	EPI_ISL_794801	17/12/2020	Europe / Italy / Apulia / Foggia	B.1.416
hCoV-19/Italy/APU-IZSPB_475PT/2020	EPI_ISL_794802	17/12/2020	Europe / Italy / Apulia / Foggia	B.1.177
hCoV-19/Italy/APU-IZSPB_476PT/2020	EPI_ISL_794803	15/12/2020	Europe / Italy / Apulia / BAT	B.1.177
hCoV-19/Italy/APU-IZSPB_477PT/2020	EPI_ISL_794804	15/12/2020	Europe / Italy / Apulia / Foggia	B.1.177
hCoV-19/Italy/APU-IZSPB_478PT/2020	EPI_ISL_794805	15/12/2020	Europe / Italy / Apulia / Foggia	B.1.177
hCoV-19/Italy/APU-IZSPB_479PT/2020	EPI_ISL_794806	15/12/2020	Europe / Italy / Apulia / Bari	B.1.177
hCoV-19/Italy/APU-IZSPB_480PT/2020	EPI_ISL_794807	15/12/2020	Europe / Italy / Apulia / Foggia	B.1.177
hCoV-19/Italy/APU-IZSPB_481PT/2020	EPI_ISL_794808	15/12/2020	Europe / Italy / Apulia / BAT	B.1.177
hCoV-19/Italy/APU-IZSPB_482PT/2020	EPI_ISL_794809	16/12/2020	Europe / Italy / Apulia / Foggia	B.1.416
hCoV-19/Italy/APU-IZSPB_483PT/2020	EPI_ISL_794810	16/12/2020	Europe / Italy / Apulia / Foggia	B.1.177
hCoV-19/Italy/APU-IZSPB_484PT/2020	EPI_ISL_794811	16/12/2020	Europe / Italy / Apulia / Foggia	B.1.177
hCoV-19/Italy/APU-IZSPB_485PT/2020	EPI_ISL_794812	17/12/2020	Europe / Italy / Apulia / Foggia	B.1.416
hCoV-19/Italy/APU-IZSPB_486PT/2020	EPI_ISL_794813	17/12/2020	Europe / Italy / Apulia / Foggia	B.1.177
hCoV-19/Italy/APU-IZSPB_487PT/2020	EPI_ISL_794814	17/12/2020	Europe / Italy / Apulia / Foggia	B.1.1.39
hCoV-19/Italy/APU-IZSPB_488PT/2020	EPI_ISL_794815	16/12/2020	Europe / Italy / Apulia / Foggia	B.1.177
hCoV-19/Italy/APU-IZSPB_489PT/2020	EPI_ISL_794816	16/12/2020	Europe / Italy / Apulia / Foggia	B.1.177
hCoV-19/Italy/APU-IZSPB_490PT/2020	EPI_ISL_794817	17/12/2020	Europe / Italy / Apulia / Foggia	B.1.177
hCoV-19/Italy/APU-IZSPB-491PT/2020	EPI_ISL_876742	29/12/2020	Europe / Italy / Apulia / Lecce / Carmiano	B.1.177
hCoV-19/Italy/APU-IZSPB_492PT/2020	EPI_ISL_918408	27/12/2020	Europe / Italy / Apulia / Lecce / Lizzanello	B.1.1.7
hCoV-19/Italy/APU-IZSPB_493PT/2020	EPI_ISL_918409	29/12/2020	Europe / Italy / Apulia / Lecce / Carmiano	B.1.1.7
hCoV-19/Italy/APU-IZSPB-498PT/2020	EPI_ISL_876743	29/12/2020	Europe / Italy / Apulia / Brindisi	B.1.160
hCoV-19/Italy/APU-IZSPB-500PT/2020	EPI_ISL_876744	28/12/2020	Europe / Italy / Apulia / Foggia	B.1.177
hCoV-19/Italy/APU-IZSPB-501PT/2020	EPI_ISL_876745	28/12/2020	Europe / Italy / Apulia / Foggia	B.1.177
hCoV-19/Italy/APU-IZSPB-502PT/2020	EPI_ISL_876746	28/12/2020	Europe / Italy / Apulia / Foggia / Serracapriola	B.1.177
hCoV-19/Italy/APU-IZSPB-503PT/2020	EPI_ISL_876747	28/12/2020	Europe / Italy / Apulia / Foggia	B.1.177
hCoV-19/Italy/APU-IZSPB-504PT/2020	EPI_ISL_876787	14/12/2020	Europe / Italy / Apulia / Foggia	B.1.177
hCoV-19/Italy/APU-IZSPB-505PT/2020	EPI_ISL_876788	14/12/2020	Europe / Italy / Apulia / Foggia	B.1.177
hCoV-19/Italy/APU-IZSPB-506PT/2020	EPI_ISL_876795	12/12/2020	Europe / Italy / Apulia / Foggia	B.1.177

hCoV-19/Italy/APU-IZSPB-507PT/2020	EPI_ISL_876789	14/12/2020	Europe / Italy / Apulia / Foggia	B.1.416
hCoV-19/Italy/APU-IZSPB-508PT/2020	EPI_ISL_876796	12/12/2020	Europe / Italy / Apulia / Foggia	B.1.221
hCoV-19/Italy/APU-IZSPB-509PT/2020	EPI_ISL_876790	14/12/2020	Europe / Italy / Apulia / Foggia	B.1.416
hCoV-19/Italy/APU-IZSPB-510PT/2020	EPI_ISL_876797	12/12/2020	Europe / Italy / Apulia / Foggia	B.1.416
hCoV-19/Italy/APU-IZSPB-511PT/2020	EPI_ISL_876791	14/12/2020	Europe / Italy / Apulia / Foggia	B.1.177
hCoV-19/Italy/APU-IZSPB-512PT/2020	EPI_ISL_876798	12/12/2020	Europe / Italy / Apulia / Foggia	B.1.177
hCoV-19/Italy/APU-IZSPB-513PT/2020	EPI_ISL_876799	12/12/2020	Europe / Italy / Apulia / Foggia	B.1.177
hCoV-19/Italy/APU-IZSPB-514PT/2020	EPI_ISL_876792	14/12/2020	Europe / Italy / Apulia / Foggia	B.1.177
hCoV-19/Italy/APU-IZSPB-515PT/2020	EPI_ISL_876793	14/12/2020	Europe / Italy / Apulia / Foggia	B.1.177
hCoV-19/Italy/APU-IZSPB-516PT/2020	EPI_ISL_876776	17/12/2020	Europe / Italy / Apulia / Lecce / Vernole	B.1.177
hCoV-19/Italy/APU-IZSPB-517PT/2020	EPI_ISL_876778	16/12/2020	Europe / Italy / Apulia / Foggia	B.1.177
hCoV-19/Italy/APU-IZSPB-518PT/2020	EPI_ISL_876777	17/12/2020	Europe / Italy / Apulia / Foggia	B.1.177
hCoV-19/Italy/APU-IZSPB-519PT/2020	EPI_ISL_876808	11/12/2020	Europe / Italy / Apulia / Foggia	B.1.177
hCoV-19/Italy/APU-IZSPB-520PT/2020	EPI_ISL_876809	11/12/2020	Europe / Italy / Apulia / Foggia	B.1.177
hCoV-19/Italy/APU-IZSPB-521PT/2020	EPI_ISL_876784	15/12/2020	Europe / Italy / Apulia / BAT / Bisceglie	B.1.177
hCoV-19/Italy/APU-IZSPB-522PT/2020	EPI_ISL_876785	15/12/2020	Europe / Italy / Apulia / Foggia	B.1.177
hCoV-19/Italy/APU-IZSPB-523PT/2020	EPI_ISL_876794	14/12/2020	Europe / Italy / Apulia / Foggia	B.1.177.8
hCoV-19/Italy/APU-IZSPB-524PT/2020	EPI_ISL_876786	15/12/2020	Europe / Italy / Apulia / Foggia	B.1.177
hCoV-19/Italy/APU-IZSPB-525PT/2020	EPI_ISL_876748	23/12/2020	Europe / Italy / Apulia / Foggia	B.1.177
hCoV-19/Italy/APU-IZSPB-526PT/2020	EPI_ISL_876749	23/12/2020	Europe / Italy / Apulia / Foggia / Mattinata	B.1.177
hCoV-19/Italy/APU-IZSPB-527PT/2020	EPI_ISL_876751	22/12/2020	Europe / Italy / Apulia / Foggia	B.1.177
hCoV-19/Italy/APU-IZSPB-528PT/2020	EPI_ISL_876750	23/12/2020	Europe / Italy / Apulia / Foggia	B.1.177
hCoV-19/Italy/APU-IZSPB-529PT/2020	EPI_ISL_876779	16/12/2020	Europe / Italy / Apulia / Foggia	B.1.177
hCoV-19/Italy/APU-IZSPB-530PT/2020	EPI_ISL_876780	16/12/2020	Europe / Italy / Apulia / Foggia	B.1.177
hCoV-19/Italy/APU-IZSPB-531PT/2020	EPI_ISL_876781	16/12/2020	Europe / Italy / Apulia / Foggia	B.1.177
hCoV-19/Italy/APU-IZSPB-532PT/2020	EPI_ISL_876782	16/12/2020	Europe / Italy / Apulia / Foggia	B.1.177
hCoV-19/Italy/APU-IZSPB-533PT/2020	EPI_ISL_876783	16/12/2020	Europe / Italy / Apulia / Foggia	B.1.177
hCoV-19/Italy/APU-IZSPB-534PT/2020	EPI_ISL_876760	20/12/2020	Europe / Italy / Apulia / Foggia	B.1.177
hCoV-19/Italy/APU-IZSPB-535PT/2020	EPI_ISL_876752	21/12/2020	Europe / Italy / Apulia / Foggia	B.1.177
hCoV-19/Italy/APU-IZSPB-536PT/2020	EPI_ISL_876761	20/12/2020	Europe / Italy / Apulia / Foggia	B.1.177
hCoV-19/Italy/APU-IZSPB-537PT/2020	EPI_ISL_876762	20/12/2020	Europe / Italy / Apulia / Foggia	B.1.177
hCoV-19/Italy/APU-IZSPB-538PT/2020	EPI_ISL_876753	21/12/2020	Europe / Italy / Apulia / Foggia	B.1.177
hCoV-19/Italy/APU-IZSPB-539PT/2020	EPI_ISL_876740	21/12/2020	Europe / Italy / Apulia / Foggia	B.1.416
hCoV-19/Italy/APU-IZSPB-540PT/2020	EPI_ISL_876763	20/12/2020	Europe / Italy / Apulia / Foggia	B.1.177
hCoV-19/Italy/APU-IZSPB-541PT/2020	EPI_ISL_876754	21/12/2020	Europe / Italy / Apulia / Foggia	B.1.177
hCoV-19/Italy/APU-IZSPB-542PT/2020	EPI_ISL_876764	20/12/2020	Europe / Italy / Apulia / Foggia	B.1.177
hCoV-19/Italy/APU-IZSPB-543PT/2020	EPI_ISL_876755	21/12/2020	Europe / Italy / Apulia / Foggia	B.1.177
hCoV-19/Italy/APU-IZSPB-544PT/2020	EPI_ISL_876756	21/12/2020	Europe / Italy / Apulia / Foggia	B.1.177
hCoV-19/Italy/APU-IZSPB-545PT/2020	EPI_ISL_876765	20/12/2020	Europe / Italy / Apulia / Foggia	B.1.177
hCoV-19/Italy/APU-IZSPB-546PT/2020	EPI_ISL_876766	20/12/2020	Europe / Italy / Apulia / Foggia	B.1.177
hCoV-19/Italy/APU-IZSPB-547PT/2020	EPI_ISL_876767	20/12/2020	Europe / Italy / Apulia / Foggia	B.1.177
hCoV-19/Italy/APU-IZSPB-548PT/2020	EPI_ISL_876768	20/12/2020	Europe / Italy / Apulia / Foggia	B.1.177
hCoV-19/Italy/APU-IZSPB-549PT/2020	EPI_ISL_876769	20/12/2020	Europe / Italy / Apulia / Foggia	B.1.177
hCoV-19/Italy/APU-IZSPB-550PT/2020	EPI_ISL_876757	21/12/2020	Europe / Italy / Apulia / Foggia	B.1.177
hCoV-19/Italy/APU-IZSPB-551PT/2020	EPI_ISL_876770	20/12/2020	Europe / Italy / Apulia / Foggia	B.1.177
hCoV-19/Italy/APU-IZSPB-552PT/2020	EPI_ISL_876771	20/12/2020	Europe / Italy / Apulia / Foggia	B.1.177
hCoV-19/Italy/APU-IZSPB-553PT/2020	EPI_ISL_876772	20/12/2020	Europe / Italy / Apulia / Foggia	B.1.177
hCoV-19/Italy/APU-IZSPB-554PT/2020	EPI_ISL_876773	20/12/2020	Europe / Italy / Apulia / Foggia	B.1.177
hCoV-19/Italy/APU-IZSPB-555PT/2020	EPI_ISL_876774	20/12/2020	Europe / Italy / Apulia / Foggia	B.1.177
hCoV-19/Italy/APU-IZSPB-557PT/2020	EPI_ISL_876758	21/12/2020	Europe / Italy / Apulia / Foggia	B.1.177
hCoV-19/Italy/APU-IZSPB-558PT/2020	EPI_ISL_876775	20/12/2020	Europe / Italy / Apulia / Foggia	B.1.177
hCoV-19/Italy/APU-IZSPB-559PT/2020	EPI_ISL_876759	21/12/2020	Europe / Italy / Apulia / Foggia	B.1.177
hCoV-19/Italy/APU-IZSPB-567PT/2020	EPI_ISL_876810	24/10/2020	Europe / Italy / Apulia / BAT / Bisceglie	B.1.1.229
hCoV-19/Italy/APU-IZSPB-568PT/2021	EPI_ISL_876741	11/01/2021	Europe / Italy / Apulia / Foggia / Peschici	B.1.177
hCoV-19/Italy/APU-IZSPB_572PT/2021	EPI_ISL_918410	23/01/2021	Europe / Italy / Apulia / Bari	B.1.1.7
hCoV-19/Italy/APU-IZSPB-142APT/2020	EPI_ISL_722896	24/06/2020	Europe / Italy / Apulia / Bari / Mola di Bari	B.1.1.162
hCoV-19/Italy/APU-IZSPB-147APT/2020	EPI_ISL_722855	20/07/2020	Europe / Italy / Apulia / Bari / Sammichele di Bari	B.1.1.1
hCoV-19/Italy/APU-IZSPB-149APT/2020	EPI_ISL_722912	2020	Europe / Italy / Apulia / Lecce / Galatina	B.1.1.316
hCoV-19/Italy/APU-IZSPB-151APT/2020	EPI_ISL_722897	28/07/2020	Europe / Italy / Apulia / Bari / Modugno	B.1.1.115

hCoV-19/Italy/APU-IZSPB-153APT/2020	EPI_ISL_722856	18/07/2020	Europe / Italy / Apulia / Brindisi / Fasano	B.1.1.305
hCoV-19/Italy/APU-IZSPB-156APT/2020	EPI_ISL_722857	18/07/2020	Europe / Italy / Apulia / Brindisi / Fasano	B.1.1.305
hCoV-19/Italy/APU-IZSPB-159APT/2020	EPI_ISL_722858	18/07/2020	Europe / Italy / Apulia / Brindisi / Fasano	B.1.1.305
hCoV-19/Italy/APU-IZSPB_169APT/2020	EPI_ISL_794745	31/03/2020	Europe / Italy / Apulia / Bari	B.1.1.74
hCoV-19/Italy/APU-IZSPB-170APT/2020	EPI_ISL_649190	17/08/2020	Europe / Italy / Apulia	B.1.1.316
hCoV-19/Italy/APU-IZSPB-172APT/2020	EPI_ISL_649191	20/08/2020	Europe / Italy / Apulia	B.1.1.229
hCoV-19/Italy/BAS-IZSPB-173APT/2020	EPI_ISL_649784	2020	Europe / Italy / Basilicata	B.1.416
hCoV-19/Italy/APU-IZSPB-177APT/2020	EPI_ISL_722851	28/08/2020	Europe / Italy / Apulia / Bari / Triggiano	B.1.1.229
hCoV-19/Italy/APU-IZSPB-179APT/2020	EPI_ISL_722852	28/08/2020	Europe / Italy / Apulia / Bari	B.1.1.229
hCoV-19/Italy/APU-IZSPB-191APT/2020	EPI_ISL_722853	28/08/2020	Europe / Italy / Apulia / Bari	B.1.1.229
hCoV-19/Italy/APU-IZSPB-193APT/2020	EPI_ISL_649940	28/08/2020	Europe / Italy / Apulia / Foggia	B.1
hCoV-19/Italy/APU-IZSPB-199APT/2020	EPI_ISL_722854	28/08/2020	Europe / Italy / Apulia / Bari	B.1.1.229
hCoV-19/Italy/APU-IZSPB-98APT/2020	EPI_ISL_649189	10/04/2020	Europe / Italy / Apulia	B.1.1.317

Table S1: Data of 298 human SARS-CoV-2 genomes collected from Apulia and Basilicata, sequenced in this study and submitted on GISAID (Accession ID).

GISAID Accession	Ns	Mapped bases	Mean coverage	Standard deviation	Number of reads	Mapped reads	Read min/max/mean length	Overlapping read pairs	Duplicated reads (flagged)	Duplicated reads (estimated)	Duplication rate	Clipped reads
EPI_ISL_876811	30	225091524	7,527.3894	55,168.7503	3,712,092	3,602,474 / 97.05%	2 / 136 / 67.04	1,665,989 / 89.76%	0 / 0%	3,588,247 / 96.66%	74.4%	613,242 / 16.52%
EPI_ISL_876812	50	181048592	6,054.5294	22,540.2039	2,085,198	2,069,110 / 99.23%	15 / 136 / 94.63	991,790 / 95.13%	0 / 0%	2,052,525 / 98.43%	74.74%	483,276 / 23.18%
EPI_ISL_876739	262	274795098	9,189.5495	82,257.4386	3,775,270	3,745,944 / 99.22%	10 / 151 / 78.85	1,868,426 / 98.98%	0 / 0%	3,740,491 / 99.08%	87.11%	672,304 / 17.81%
EPI_ISL_876813	44	200220360	6,695.6613	14,861.9755	2,302,438	1,796,246 / 78.01%	14 / 136 / 107.27	886,648 / 77.02%	0 / 0%	1,785,287 / 77.54%	65.89%	168,987 / 7.34%
EPI_ISL_649189	4	139945680	4,679.988	2,468.0339	708,510	706,964 / 99.78%	16 / 236 / 198.83	339,011 / 95.7%	0 / 0%	679,388 / 95.89%	91.08%	21,394 / 3.02%
EPI_ISL_722896	3	133719733	4,471.7832	2,094.407	709,430	707,633 / 99.75%	14 / 236 / 189.51	352,478 / 99.37%	0 / 0%	681,415 / 96.05%	88.99%	17,466 / 2.46%
EPI_ISL_722855	29	74202288	2,481.4329	2,335.0135	440,630	383,537 / 87.04%	10 / 236 / 192.85	190,509 / 86.47%	0 / 0%	362,212 / 82.2%	80.89%	11,686 / 2.65%
EPI_ISL_722912	2	104479918	3,493.9611	1,601.9464	582,626	580,285 / 99.6%	12 / 236 / 180.55	289,536 / 99.39%	0 / 0%	554,789 / 95.22%	87.59%	13,122 / 2.25%
EPI_ISL_722899	2	109275729	3,654.34	2,054.2373	583,122	561,106 / 96.22%	15 / 236 / 195.37	279,520 / 95.87%	0 / 0%	536,146 / 91.94%	86.1%	16,887 / 2.9%
EPI_ISL_722897	0	93070221	3,112.4041	1,314.0962	509,838	508,219 / 99.68%	17 / 236 / 183.89	250,778 / 98.38%	0 / 0%	482,119 / 94.56%	87.49%	14,788 / 2.9%
EPI_ISL_722856	63	82982123	2,775.0434	2,399.1444	487,268	425,053 / 87.23%	17 / 236 / 194.77	211,426 / 86.78%	0 / 0%	402,855 / 82.68%	82.02%	13,958 / 2.86%
EPI_ISL_722857	2	118795206	3,972.6852	1,998.6194	644,474	638,626 / 99.09%	14 / 236 / 186.75	318,069 / 98.71%	0 / 0%	612,467 / 95.03%	88.43%	20,603 / 3.2%
EPI_ISL_722872	11	118034579	3,947.2487	1,802.9499	626,43	624,551 / 99.7%	16 / 236 / 189.59	310,786 / 99.22%	0 / 0%	598,515 / 95.54%	88.18%	15,514 / 2.48%
EPI_ISL_722858	47	108381334	3,624.4301	2,256.6105	591,828	573,813 / 96.96%	15 / 236 / 189.35	286,049 / 96.67%	0 / 0%	549,308 / 92.82%	85.88%	13,810 / 2.33%
EPI_ISL_794745	7	88007278	2,943.0919	2,105.8589	492,798	441,695 / 89.63%	7 / 251 / 193.08	212,735 / 86.34%	0 / 0%	420,455 / 85.32%	85.23%	32,027 / 6.5%
EPI_ISL_649190	0	128942055	4,312.0107	1,873.8782	639,436	638,049 / 99.78%	14 / 236 / 202.64	301,441 / 94.28%	0 / 0%	609,934 / 95.39%	92.39%	16,624 / 2.6%
EPI_ISL_653823	3	112967855	3,777.8101	1,574.0779	555,246	554,335 / 99.84%	14 / 236 / 204.41	266,738 / 96.08%	0 / 0%	526,634 / 94.85%	91.3%	13,138 / 2.37%
EPI_ISL_649191	2	133364854	4,459.9155	2,271.2381	675,802	674,136 / 99.75%	16 / 236 / 198.82	327,112 / 96.81%	0 / 0%	646,029 / 95.59%	92.53%	26,206 / 3.88%
EPI_ISL_649784	0	148575038	4,968.5663	2,563.6788	746,894	745,463 / 99.81%	15 / 236 / 199.76	360,591 / 96.56%	0 / 0%	717,929 / 96.12%	91.57%	18,569 / 2.49%
EPI_ISL_722851	4	95157777	3,182.2151	1,472.8847	503,300	501,273 / 99.6%	16 / 236 / 190.37	249,002 / 98.95%	0 / 0%	475,802 / 94.54%	86.73%	12,791 / 2.54%
EPI_ISL_722859	16	82375406	2,754.7539	1,215.6234	418,370	417,319 / 99.75%	14 / 236 / 197.87	203,785 / 97.42%	0 / 0%	391,913 / 93.68%	85.55%	9,989 / 2.39%
EPI_ISL_722852	16	94148660	3,148.4687	1,491.6368	492,898	490,522 / 99.52%	16 / 236 / 192.5	243,634 / 98.86%	0 / 0%	465,229 / 94.39%	86.24%	12,224 / 2.48%
EPI_ISL_722898	3	102220434	3,418.4006	1,643.4721	535,430	533,240 / 99.59%	15 / 236 / 192.49	264,432 / 98.77%	0 / 0%	507,121 / 94.71%	87.95%	19,813 / 3.7%
EPI_ISL_722860	16	101602984	3,397.7522	1,571.1477	525,604	523,843 / 99.66%	16 / 236 / 194.53	258,380 / 98.32%	0 / 0%	497,998 / 94.75%	86.97%	13,287 / 2.53%
EPI_ISL_649785	29	41385249	1,383.9832	9,799.2031	794,512	646,318 / 81.35%	6 / 136 / 69.4	304,439 / 76.64%	0 / 0%	639,280 / 80.46%	66.79%	102,921 / 12.95%
EPI_ISL_722862	16	101997621	3,410.9494	1,538.4809	544,408	536,564 / 98.56%	14 / 236 / 191.37	263,221 / 96.7%	0 / 0%	509,744 / 93.63%	89.06%	24,641 / 4.53%
EPI_ISL_722863	10	104897245	3,507.9171	1,569.0165	544,430	542,898 / 99.72%	14 / 236 / 193.92	269,968 / 99.17%	0 / 0%	516,770 / 94.92%	87.82%	19,305 / 3.55%
EPI_ISL_722864	2	93930235	3,141.1643	1,418.6822	498,064	491,293 / 98.64%	16 / 236 / 191.83	244,590 / 98.22%	0 / 0%	466,005 / 93.56%	85.94%	13,584 / 2.73%
EPI_ISL_722895	3	126501814	4,230.4054	1,922.9264	672,55	670,610 / 99.71%	15 / 236 / 189.04	333,558 / 99.19%	0 / 0%	644,265 / 95.79%	88.84%	14,847 / 2.21%
EPI_ISL_722865	1	115843088	3,873.9621	2,077.0528	622,376	614,129 / 98.67%	14 / 236 / 189.09	304,537 / 97.86%	0 / 0%	588,433 / 94.55%	87.34%	15,878 / 2.55%
EPI_ISL_722866	24	113607763	3,799.2095	2,052.7834	636,688	614,838 / 96.57%	15 / 236 / 185.34	305,369 / 95.92%	0 / 0%	589,050 / 92.52%	87.69%	15,740 / 2.47%
EPI_ISL_649938	19	91623470	3,064.0227	19,436.7675	1,250,670	1,211,180 / 96.84%	2 / 136 / 81.86	553,153 / 88.46%	0 / 0%	1,203,063 / 96.19%	73.66%	377,490 / 30.18%
EPI_ISL_722853	7	119571401	3,998.6423	1,866.1171	609,092	607,309 / 99.71%	16 / 236 / 197.55	302,116 / 99.2%	0 / 0%	581,204 / 95.42%	87.93%	19,160 / 3.15%
EPI_ISL_649939	25	21330430	7133207	3,335.5042	288,532	279,846 / 96.99%	3 / 136 / 80.69	122,390 / 84.84%	0 / 0%	273,692 / 94.86%	65.79%	43,188 / 14.97%
EPI_ISL_649940	7	202821692	6,782.6536	3,261.7493	1,073,914	1,071,226 / 99.75%	13 / 236 / 189.97	528,737 / 98.47%	0 / 0%	1,043,234 / 97.14%	92.63%	27,884 / 2.6%
EPI_ISL_722892	1	93461086	3,125.4752	1,435.7568	459,420	457,441 / 99.57%	14 / 236 / 205.27	216,853 / 94.4%	0 / 0%	429,941 / 93.58%	90.69%	21,845 / 4.75%
EPI_ISL_722867	4	104330154	3,488.9527	1,597.5581	532,992	531,763 / 99.77%	15 / 236 / 196.82	263,848 / 99.01%	0 / 0%	505,979 / 94.93%	87.38%	16,194 / 3.04%
EPI_ISL_722900	0	94718246	3,167.5165	1,348.6075	530,196	525,557 / 99.13%	12 / 236 / 180.91	259,507 / 97.89%	0 / 0%	498,977 / 94.11%	88.32%	17,338 / 3.27%
EPI_ISL_722868	3	115110096	3,849.4498	1,727.2627	609,088	606,952 / 99.65%	17 / 236 / 190.18	302,549 / 99.34%	0 / 0%	581,047 / 95.4%	88.26%	16,048 / 2.63%
EPI_ISL_653763	119	98560399	3,296.0037	2,972.0266	775,504	770,847 / 99.4%	18 / 136 / 128.66	372,284 / 96.01%	0 / 0%	759,927 / 97.99%	56.25%	36,112 / 4.66%
EPI_ISL_722854	1	98592150	3,297.0655	1,441.435	509,456	507,994 / 99.71%	14 / 236 / 194.88	252,015 / 98.93%	0 / 0%	481,765 / 94.56%	87.51%	18,653 / 3.66%
EPI_ISL_653764	60	29634196	991.0108	1,080.9075	248,534	247,191 / 99.46%	17 / 136 / 125.04	97,919 / 78.8%	0 / 0%	237,622 / 95.61%	50.53%	33,966 / 13.67%
EPI_ISL_722869	5	111133715	3,716.4738	1,646.411	591,514	588,569 / 99.5%	15 / 236 / 189.27	291,915 / 98.7%	0 / 0%	562,346 / 95.07%	88.52%	14,859 / 2.51%
EPI_ISL_722870	14	117187839	3,918.9325	1,731.8867	635,232	630,688 / 99.28%	16 / 236 / 186.35	312,226 / 98.3%	0 / 0%	603,804 / 95.05%	89.65%	18,358 / 2.89%
EPI_ISL_722871	12	77711842	2,598.7975	1,354.8182	384,472	383,533 / 99.76%	15 / 236 / 203.3	184,289 / 95.87%	0 / 0%	357,644 / 93.02%	86.16%	13,153 / 3.42%
EPI_ISL_653784	5	41918731	1,401.8236	6738664	200,124	199,667 / 99.77%	18 / 236 / 210.68	85,827 / 85.77%	0 / 0%	175,887 / 87.89%	79.88%	5,834 / 2.92%
EPI_ISL_653785	41	185358683	6,198.6651	4,399.9459	985,856	970,646 / 98.46%	11 / 236 / 191.37	480,937 / 97.57%	0 / 0%	944,343 / 95.79%	89.67%	25,824 / 2.62%
EPI_ISL_653786	1	113121362	3,782.9436	1,646.4922	565,044	563,486 / 99.72%	15 / 236 / 201.51	267,039 / 94.52%	0 / 0%	535,692 / 94.81%	0,92	18,887 / 3.34%
EPI_ISL_653765	3	210939116	7,054.1122	3,631.9819	1,211,263	1,204,669 / 99.46%	92.55%	21,979 / 1.81%				
EPI_ISL_653766	45	227702029	7,614.6885	5,739.4081	1,722,650	1,210,078 / 70.25%	14 / 236 / 195.54	600,532 / 69.72%	0 / 0%	1,184,072 / 68.74%	90%	27,322 / 1.59%
EPI_ISL_653767	0	377627310	12,628.4089	5,833.2701	2,041,206	2,035,397 / 99.72%	13 / 236 / 185.89	1,007,098 / 98.68%	0 / 0%	2,006,552 / 98.3%	96.08%	46,717 / 2.29%
EPI_ISL_653768	8	207563712	6,941.2337	3,478.5351	1,273,837	1,267,375 / 99.49%	93.56%	20,972 / 1.65%				
EPI_ISL_653769	0	381264409	12,750.0388	5,840.0441	2,010,208	2,004,311 / 99.71%	8 / 236 / 190.72	985,803 / 98.08%	0 / 0%	1,975,309 / 98.26%	96.33%	48,873 / 2.43%
EPI_ISL_653770	3	382971397	12,807.1229	7,195.0717	2,258,968	2,242,498 / 99.27%	13 / 236 / 171.37	1,110,967 / 98.36%	0 / 0%	2,213,697 / 98%	95.98%	67,620 / 2.99%

EPI_ISL_653771	41	321251918	10,743.1334	8,441.5249	1,780,828	1,721,239 / 96.65%	9 / 236 / 186.97	854,142 / 95.93%	0 / 0%	1,693,901 / 95.12%	92.67%	56,004 / 3.14%
EPI_ISL_653772	0	398470207	13,325.4258	7,574.4933	2,177,110	2,157,567 / 99.1%	14 / 236 / 185.16	1,071,884 / 98.47%	0 / 0%	2,129,122 / 97.8%	94.95%	57,092 / 2.62%
EPI_ISL_653773	37	401275647	13,419.2438	6,760.6237	2,138,834	2,125,078 / 99.36%	9 / 236 / 189.14	1,058,157 / 98.95%	0 / 0%	2,096,465 / 98.02%	95.23%	41,751 / 1.95%
EPI_ISL_653774	11	345501047	11,554.0597	5,792.4882	1,772,298	1,767,931 / 99.75%	11 / 236 / 195.95	878,786 / 99.17%	0 / 0%	1,739,432 / 98.15%	94.8%	45,554 / 2.57%
EPI_ISL_653775	10	392787389	13,135.384	7,018.3014	2,046,278	2,017,516 / 98.59%	14 / 236 / 195.11	1,003,628 / 98.09%	0 / 0%	1,989,190 / 97.21%	94.46%	46,771 / 2.29%
EPI_ISL_653776	1	340936676	11,401.4205	5,359.4676	1,953,986	1,947,836 / 99.69%	13 / 236 / 175.48	967,875 / 99.07%	0 / 0%	1,918,936 / 98.21%	95.97%	50,162 / 2.57%
EPI_ISL_653777	38	401303016	13,420.159	9,860.0356	2,157,328	2,132,453 / 98.85%	9 / 236 / 188.54	1,058,957 / 98.17%	0 / 0%	2,104,662 / 97.56%	93.88%	58,965 / 2.73%
EPI_ISL_653778	9	408037365	13,645.3655	7,374.4161	2,128,542	2,123,053 / 99.74%	14 / 236 / 192.67	1,055,508 / 99.18%	0 / 0%	2,094,457 / 98.4%	95.33%	58,421 / 2.74%
EPI_ISL_653779	1	431259402	14,421.9444	7,005.0652	2,338,188	2,327,324 / 99.54%	13 / 236 / 185.71	1,152,811 / 98.61%	0 / 0%	2,298,408 / 98.3%	96.11%	52,421 / 2.24%
EPI_ISL_653780	9	359263463	12,014.295	6,265.3328	2,067,412	1,929,223 / 93.32%	11 / 236 / 188.42	955,783 / 92.46%	0 / 0%	1,900,899 / 91.95%	94.83%	39,831 / 1.93%
EPI_ISL_653781	0	333207763	11,142.9543	5,807.4258	1,852,416	1,759,026 / 94.96%	12 / 236 / 190.87	875,451 / 94.52%	0 / 0%	1,730,815 / 93.44%	94.2%	38,980 / 2.1%
EPI_ISL_653782	4	566436931	18,942.4784	9,487.5634	3,092,292	2,952,117 / 95.47%	13 / 236 / 193.17	1,465,762 / 94.8%	0 / 0%	2,923,134 / 94.53%	96.63%	70,423 / 2.28%
EPI_ISL_653783	41	513532404	17,173.2737	10,344.1165	2,944,684	2,670,074 / 90.67%	11 / 236 / 194.89	1,327,822 / 90.18%	0 / 0%	2,641,479 / 89.7%	95.97%	86,117 / 2.92%
EPI_ISL_653787	72	102990146	3,444.1409	3,651.8162	720,614	519,565 / 72.1%	15 / 236 / 196.6	257,297 / 71.41%	0 / 0%	497,366 / 69.02%	82.22%	18,583 / 2.58%
EPI_ISL_653788	60	90923529	3,040.6156	3,211.0785	713,182	468,294 / 65.66%	15 / 236 / 191.83	232,240 / 65.13%	0 / 0%	447,153 / 62.7%	81.17%	14,803 / 2.08%
EPI_ISL_653789	13	202599636	6,775.2278	3,533.8635	1,034,934	1,029,633 / 99.49%	13 / 236 / 197.49	511,013 / 98.75%	0 / 0%	1,002,295 / 96.85%	91.24%	30,672 / 2.96%
EPI_ISL_653790	41	186964266	6,252.3582	6,217.6596	1,158,394	991,976 / 85.63%	16 / 236 / 186.87	490,250 / 84.64%	0 / 0%	967,336 / 83.51%	87.21%	26,988 / 2.33%
EPI_ISL_653791	0	147129582	4,920.2281	2,727.2738	796,326	757,318 / 95.1%	14 / 236 / 195.31	372,594 / 93.58%	0 / 0%	730,589 / 91.74%	89.7%	16,594 / 2.08%
EPI_ISL_653792	2	194092391	6,490.7331	3,336.3691	988,850	983,457 / 99.45%	14 / 236 / 198.11	488,620 / 98.83%	0 / 0%	956,445 / 96.72%	90.64%	29,256 / 2.96%
EPI_ISL_653793	5	231577761	7,744.2986	4,199.2333	1,202,426	1,195,863 / 99.45%	15 / 236 / 194.16	595,519 / 99.05%	0 / 0%	1,168,772 / 97.2%	91.3%	29,751 / 2.47%
EPI_ISL_653794	4	206708426	6,912.6317	3,790.0717	1,083,088	1,071,258 / 98.91%	16 / 236 / 193.69	532,765 / 98.38%	0 / 0%	1,044,258 / 96.41%	90.8%	31,772 / 2.93%
EPI_ISL_653795	44	94231941	3,151.2538	3,327.8032	763,944	504,949 / 66.1%	15 / 236 / 184.53	250,280 / 65.52%	0 / 0%	483,581 / 63.3%	82.17%	14,577 / 1.91%
EPI_ISL_653796	5	225275997	7,533.5584	4,311.0311	1,202,190	1,172,836 / 97.56%	13 / 236 / 192.62	582,992 / 96.99%	0 / 0%	1,145,720 / 95.3%	91.04%	27,319 / 2.27%
EPI_ISL_653797	3	178420406	5,966.639	2,973.8078	920,302	917,570 / 99.7%	12 / 236 / 195.19	456,017 / 99.1%	0 / 0%	890,448 / 96.76%	90.53%	25,854 / 2.81%
EPI_ISL_653798	12	173836669	5,813.3521	3,178.5933	883,140	880,681 / 99.72%	15 / 236 / 197.96	428,847 / 97.12%	0 / 0%	853,180 / 96.61%	91.42%	20,925 / 2.37%
EPI_ISL_653799	43	94466932	3,159.1122	3,413.526	843,774	510,350 / 60.48%	11 / 236 / 185.45	253,399 / 60.06%	0 / 0%	488,514 / 57.9%	82.08%	18,983 / 2.25%
EPI_ISL_653800	43	195411115	6,534.8331	3,756.865	1,070,690	1,049,094 / 97.98%	16 / 236 / 186.57	523,151 / 97.72%	0 / 0%	1,022,416 / 95.49%	90.59%	21,434 / 2%
EPI_ISL_653801	50	127472486	4,262.8661	2,817.3238	927,120	681,514 / 73.51%	15 / 236 / 192.88	339,302 / 73.19%	0 / 0%	656,635 / 70.83%	86.97%	13,938 / 1.5%
EPI_ISL_653802	3	156742315	5,241.692	2,479.4335	867,096	863,908 / 99.63%	15 / 236 / 181.9	427,968 / 98.71%	0 / 0%	836,625 / 96.49%	91.03%	19,096 / 2.2%
EPI_ISL_653803	35	166455978	5,566.5311	3,896.6614	914,794	875,583 / 95.71%	12 / 236 / 190.25	433,742 / 94.83%	0 / 0%	849,579 / 92.87%	88.73%	22,997 / 2.51%
EPI_ISL_653804	1	178602163	5,972.7172	2,927.2662	914,436	909,853 / 99.5%	10 / 236 / 197	452,259 / 98.92%	0 / 0%	882,810 / 96.54%	91.11%	26,078 / 2.85%
EPI_ISL_653805	3	153119933	5,120.5542	2,541.1633	776,740	774,487 / 99.71%	16 / 236 / 198.46	381,449 / 98.22%	0 / 0%	747,578 / 96.25%	89.67%	22,336 / 2.88%
EPI_ISL_653806	59	172876358	5,781.2379	4,369.9229	941,788	893,207 / 94.84%	9 / 236 / 194.11	442,754 / 94.02%	0 / 0%	867,263 / 92.09%	88.88%	35,356 / 3.75%
EPI_ISL_653807	4	176018151	5,886.3041	2,947.6212	950,896	916,320 / 96.36%	15 / 236 / 193.14	451,626 / 94.99%	0 / 0%	888,943 / 93.48%	91.2%	21,521 / 2.26%
EPI_ISL_653808	12	152333391	5,094.2511	2,492.6478	817,882	796,635 / 97.4%	14 / 236 / 192.12	394,758 / 96.53%	0 / 0%	770,008 / 94.15%	89.59%	16,212 / 1.98%
EPI_ISL_653809	30	161853647	5,412.6224	4,133.897	1,034,606	831,712 / 80.39%	14 / 236 / 197.69	413,704 / 79.97%	0 / 0%	806,960 / 78%	87.56%	19,440 / 1.88%
EPI_ISL_653810	0	185628983	6,207.7043	3,211.2489	1,027,684	946,031 / 92.05%	12 / 236 / 198.2	469,612 / 91.39%	0 / 0%	919,269 / 89.45%	90.33%	21,580 / 2.1%
EPI_ISL_653811	42	223182398	7,463.5454	3,958.3562	1,217,172	1,139,852 / 93.65%	14 / 236 / 197.35	567,474 / 93.24%	0 / 0%	1,113,031 / 91.44%	91.44%	24,243 / 1.99%
EPI_ISL_653812	2	163465305	5,466.5186	3,084.7736	811,36	802,301 / 98.88%	16 / 236 / 204.3	387,309 / 95.47%	0 / 0%	774,492 / 95.46%	92.45%	20,630 / 2.54%
EPI_ISL_653813	1	169229355	5,659.2768	3,049.946	868,71	866,385 / 99.73%	14 / 236 / 196.22	424,283 / 97.68%	0 / 0%	838,938 / 96.57%	91.38%	28,548 / 3.29%
EPI_ISL_653814	4	232553605	7,776.9322	4,370.7628	1,200,774	1,183,347 / 98.55%	9 / 236 / 196.91	573,540 / 95.53%	0 / 0%	1,155,318 / 96.21%	93.22%	27,726 / 2.31%
EPI_ISL_653815	8	183917470	6,150.4688	4,028.5239	956,078	934,304 / 97.72%	13 / 236 / 197.28	464,898 / 97.25%	0 / 0%	908,296 / 95%	89.43%	23,858 / 2.5%
EPI_ISL_653816	4	201964274	6,753.9803	3,278.347	1,092,058	1,088,097 / 99.64%	15 / 236 / 186.07	542,449 / 99.34%	0 / 0%	1,060,790 / 97.14%	91.94%	21,801 / 2%
EPI_ISL_653817	14	188460804	6,302.4046	3,696.2744	960,69	951,663 / 99.06%	12 / 236 / 198.6	473,033 / 98.48%	0 / 0%	925,173 / 96.3%	89.67%	23,918 / 2.49%
EPI_ISL_653818	1	212661910	7,111.7249	3,478.3444	1,107,124	1,103,820 / 99.7%	11 / 236 / 193.3	548,739 / 99.13%	0 / 0%	1,076,222 / 97.21%	92.36%	28,243 / 2.55%
EPI_ISL_653819	2	152618103	5,103.7723	2,679.231	821,39	818,491 / 99.65%	13 / 236 / 187.21	398,627 / 97.06%	0 / 0%	790,746 / 96.27%	91.76%	25,401 / 3.09%
EPI_ISL_653820	2	144640811	4,837	2,373.5891	746,604	744,369 / 99.7%	16 / 236 / 194.94	367,170 / 98.36%	0 / 0%	717,444 / 96.09%	89.98%	20,880 / 2.8%
EPI_ISL_653821	15	203237316	6,796.5527	3,130.4079	1,020,550	1,015,923 / 99.55%	13 / 236 / 200.63	485,677 / 95.18%	0 / 0%	987,621 / 96.77%	93.6%	29,544 / 2.89%
EPI_ISL_653822	1	237053727	7,927.4229	3,894.7764	1,245,402	1,239,794 / 99.55%	13 / 236 / 191.79	615,469 / 98.84%	0 / 0%	1,212,121 / 97.33%	92.57%	29,038 / 2.33%
EPI_ISL_722908	0	46993207	1,571.5215	6059739	251,39	250,336 / 99.58%	12 / 236 / 188.58	112,102 / 89.19%	0 / 0%	224,549 / 89.32%	84.43%	9,583 / 3.81%
EPI_ISL_722911	3	81762559	2,734.2594	1,148.1314	416,908	415,384 / 99.63%	7 / 236 / 197.91	198,307 / 95.13%	0 / 0%	388,068 / 93.08%	89.37%	19,165 / 4.6%
EPI_ISL_722893	1	82178432	2,748.1668	1,053.4511	425,81	424,289 / 99.64%	13 / 236 / 194.8	203,032 / 95.36%	0 / 0%	396,914 / 93.21%	89.67%	18,752 / 4.4%
EPI_ISL_722894	25	71398834	2,387.6813	8616725	369,48	368,030 / 99.61%	16 / 236 / 194.5	175,378 / 94.93%	0 / 0%	341,620 / 92.46%	87.33%	8,451 / 2.29%
EPI_ISL_722909	0	103563543	3,463.3162	1,191.2425	573,862	572,653 / 99.79%	13 / 236 / 181.84	282,078 / 98.31%	0 / 0%	544,609 / 94.9%	92.5%	20,905 / 3.64%
EPI_ISL_722910	12	100417550	3,358.1096	1,403.0645	519,55	516,657 / 99.44%	15 / 236 / 194.92	252,570 / 97.23%	0 / 0%	489,756 / 94.27%	89.35%	14,841 / 2.86%
EPI_ISL_722901	11	76153734	2,546.6921	1,178.8716	399,51	397,904 / 99.6%	12 / 236 / 192.24	198,112 / 99.18%	0 / 0%	373,240 / 93.42%	84.65%	13,368 / 3.35%
EPI_ISL_722873	5	87815683	2,936.6847	1,465.4578	493,936	491,994 / 99.61%	13 / 236 / 179.01	241,704 / 97.87%	0 / 0%	465,664 / 94.28%	88.32%	13,398 / 2.71%
EPI_ISL_722874	3	118924324	3,977.0031	2,002.1873	619,914	614,126 / 99.07%	8 / 236 / 194.98	305,195 / 98.46%	0 / 0%	587,736 / 94.81%	88.62%	32,982 / 5.32%
EPI_ISL_722875	2	101967385	3,409.9383	1,636.9946	523,034	521,257 / 99.66%	16 / 236 / 196.37	257,615 / 98.51%	0 / 0%	495,045 / 94.65%	87.96%	18,065 / 3.45%
EPI_ISL_722876	12	85409984	2,856.2346	1,462.3482	460,046	458,307 / 99.62%	14 / 236 / 187.18	225,127 / 97.87%	0 / 0%	432,008 / 93.91%	87.74%	16,689 / 3.63%
EPI_ISL_722913	33	113611789	3,799.3442	2,091.9309	600,696	592,390 / 98.62%	14 / 236 / 192.15	295,335 / 98.33%	0 / 0%	567,590 / 94.49%	86.55%	12,522 / 2.08%
EPI_ISL_722914	9	97904918	3,274.0835	1,664.8185	514,234	509,330 / 99.05%	15 / 236 / 192.73	253,777 / 98.7%	0 / 0%	484,494 / 94.22%	85.86%	13,485 / 2.62%
EPI_ISL_722902	17	63807411	2,133.813	1,243.8182	339,378	336,629 / 99.19%	13 / 236 / 190.32	167,755 / 98.86%	0 / 0%	313,416 / 92.35%	82.45%	11,313 / 3.33%
EPI_ISL_722903	0	121023794	4,047.2125	1,902.668	672,494	670,590 / 99.72%	14 / 236 / 181.13	333,167 / 99.08%	0 / 0%	643,782 / 95.73%	89.82%	20,222 / 3.01%
EPI_ISL_722877	22	95664152	3,199.149	1,431.9965	497,864	495,037 / 99.43%	17 / 236 / 193.68	244,763 / 98.33%	0 / 0%	469,141 / 94.23%	87.55%	12,889 / 2.59%
EPI_ISL_722878	32	106158628	3,550.0996	1,847.2391	588,966	583,353 / 99.05%	14 / 236 / 182.32	290,611 / 98.69%	0 / 0%	557,915 / 94.73%	87.19%	12,766 / 2.17%
EPI_ISL_722915	33	127229336	4,254.7348	2,199.5114	671,65	665,815 / 99.13%	15 / 236 / 191.74	332,116 / 98.9%	0 / 0%	640,351 / 95.34%	87.68%	17,081 / 2.54%
EPI_ISL_722916	2	100242271	3,352.248	1,669.6316	510,898	509,082 / 99.64%	17 / 236 / 197.58	251,662 / 98.52%	0 / 0%	483,412 / 94.62%	86.85%	14,079 / 2.76%
EPI_ISL_722879	2	113252396	3,787.3256	1,822.9911	611,848	610,514 / 99.78%	16 / 236 / 186.16	304,187 / 99.43%	0 / 0%	584,361 / 95.51%	88.8%	16,682 / 2.73%
EPI_ISL_722904	33	99569468	3,329.7485	1,675.344	521,042	519,486 / 99.7%	11 / 236 / 192.24	258,443 / 99.2%	0 / 0%	494,086 / 94.83%	86.76%	14,560 / 2.79%
EPI_ISL_722917	9	97067399	3,246.0756	1,599.3235	498,358	497,251 / 99.78%	14 / 236 / 195.99	247,085 / 99.16%	0 / 0%	471,919 / 94.69%	86.54%	17,028 / 3.42%
EPI_ISL_722905	34	111735937	3,736.6129	2,072.865	630,38	606,574 / 96.22%	15 / 236 / 184.83	302,249 / 95.89%	0 / 0%	580,976 / 92.16%	87.61%	16,846 / 2.67%
EPI_ISL_722880	4	111025784	3,712.8644	1,844.2536	583,806	582,109 / 99.71%	16 / 236 / 191.66	289,818 / 99.29%	0 / 0%	555,905 / 95.22%	88.45%	23,460 / 4.02%
EPI_ISL_722881	8	55561175	1,858.0469	999.6501	284,210	283,586 / 99.78%	16 / 236 / 196.34	139,825 / 98.4%	0 / 0%	260,147 / 91.53%	81.22%	6,795 / 2.39%
EPI_ISL_722918	5	105184227	3,517.5142	1,783.169	537,520	533,496 / 99.25%	14 / 236 / 197.84	264,230 / 98.31%	0 / 0%	507,482 / 94.41%	87.92%	17,128 / 3.19%
EPI_ISL_722919	3	107763849	3,603.7805	1,579.4187	578,504	576,875 / 99.72%	12 / 236 / 187.32	286,225 / 98.95%	0 / 0%	550,605 / 95.18%	88.54%	15,199 / 2.63%
EPI_ISL_722906	6	123747153	4,138.2856	2,545.282	664,932	646,638 / 97.25%	12 / 236 / 191.9	321,847 / 96.81%	0 / 0%	621,348 / 93.45%	87.5%	20,401 / 3.07%

EPI_ISL_722882	5	105151267	3,516.412	1,607.9531	551,620	544,963 / 98.79%	17 / 236 / 193.55	271,259 / 98.35%	0 / 0%	519,267 / 94.13%	87.03%	14,937 / 2.71%
EPI_ISL_722883	3	109408538	3,658.7813	1,619.5892	562,204	560,635 / 99.72%	11 / 236 / 195.93	277,234 / 98.62%	0 / 0%	534,311 / 95.04%	88.8%	19,711 / 3.51%
EPI_ISL_722907	17	109308073	3,655.4216	1,588.1097	584,216	581,982 / 99.62%	8 / 236 / 188.58	289,983 / 99.27%	0 / 0%	555,915 / 95.16%	87.89%	18,761 / 3.21%
EPI_ISL_722884	25	116197033	3,885.7985	1,804.9558	612,636	604,931 / 98.74%	6 / 236 / 193.37	300,477 / 98.09%	0 / 0%	578,316 / 94.4%	89%	30,193 / 4.93%
EPI_ISL_722885	11	109445130	3,660.005	1,486.5758	586,942	585,471 / 99.75%	3 / 236 / 187.34	289,197 / 98.54%	0 / 0%	558,949 / 95.23%	89.05%	14,019 / 2.39%
EPI_ISL_722886	26	103126245	3,448.6923	1,589.1751	567,852	565,366 / 99.56%	14 / 236 / 182.79	281,846 / 99.27%	0 / 0%	539,707 / 95.04%	87.16%	13,076 / 2.3%
EPI_ISL_722920	41	116370310	3,891.5932	2,028.6284	605,062	594,545 / 98.26%	16 / 236 / 196.21	295,829 / 97.78%	0 / 0%	569,345 / 94.1%	86.62%	16,328 / 2.7%
EPI_ISL_722921	31	103972868	3,477.0046	3,151.653	630,424	579,192 / 91.87%	13 / 236 / 179.15	287,993 / 91.36%	0 / 0%	555,742 / 88.15%	84.63%	19,363 / 3.07%
EPI_ISL_722887	2	122648866	4,101.5572	1,823.1545	632,696	631,148 / 99.76%	9 / 236 / 195.07	309,270 / 97.76%	0 / 0%	604,274 / 95.51%	89.7%	19,477 / 3.08%
EPI_ISL_722888	3	131503289	4,397.6621	2,272.1735	693,496	689,923 / 99.48%	13 / 236 / 191.26	343,251 / 98.99%	0 / 0%	663,645 / 95.7%	89.12%	21,717 / 3.13%
EPI_ISL_722889	55	68241964	2,282.111	2,201.3779	440,722	347,656 / 78.88%	16 / 236 / 195.69	173,093 / 78.55%	0 / 0%	327,158 / 74.23%	80.46%	12,927 / 2.93%
EPI_ISL_722890	16	109001931	3,645.1838	2,067.9934	578,846	577,570 / 99.78%	13 / 236 / 189.2	287,959 / 99.49%	0 / 0%	552,712 / 95.49%	86.21%	13,826 / 2.39%
EPI_ISL_722922	29	104443451	3,492.7416	1,923.1198	604,842	591,344 / 97.77%	14 / 236 / 176.95	293,904 / 97.18%	0 / 0%	565,503 / 93.5%	87.84%	17,155 / 2.84%
EPI_ISL_722923	41	98273058	3,286.3946	1,587.6187	573,296	565,461 / 98.63%	11 / 236 / 174.49	278,913 / 97.3%	0 / 0%	539,154 / 94.04%	88.59%	17,262 / 3.01%
EPI_ISL_722924	31	207710315	6,946.1363	5,117.2493	1,147,770	1,054,006 / 91.83%	15 / 236 / 197.18	524,943 / 91.47%	0 / 0%	1,028,213 / 89.58%	88.71%	32,105 / 2.8%
EPI_ISL_722925	37	129373749	4,326.4471	3,378.2918	688,978	675,437 / 98.03%	15 / 236 / 191.42	336,989 / 97.82%	0 / 0%	651,264 / 94.53%	86.39%	18,612 / 2.7%
EPI_ISL_722891	46	128220450	4,287.8791	3,437.3381	807,286	689,419 / 85.4%	15 / 236 / 185.93	343,822 / 85.18%	0 / 0%	665,275 / 82.41%	86.46%	20,912 / 2.59%
EPI_ISL_745193	0	2011981588	67,283.6033	34,434.6709	10,029,230	10,011,111 / 99.82%	5 / 251 / 201.18	4,826,108 / 96.24%	0 / 0%	9,981,727 / 99.53%	98.67%	418,949 / 4.18%
EPI_ISL_745192	0	2446589552	81,817.5284	38,308.6986	12,244,876	12,225,116 / 99.84%	5 / 251 / 200.3	6,061,070 / 99%	0 / 0%	12,196,014 / 99.6%	98.49%	378,777 / 3.09%
EPI_ISL_794746	37	128199465	4,287.1774	4,364.546	676,23	653,607 / 96.65%	8 / 251 / 195.14	323,148 / 95.57%	0 / 0%	634,155 / 93.78%	87.89%	23,397 / 3.46%
EPI_ISL_794747	10	127464866	4,262.6113	1,923.341	695,63	694,229 / 99.8%	5 / 251 / 183.82	343,738 / 98.83%	0 / 0%	671,306 / 96.5%	89.81%	28,397 / 4.08%
EPI_ISL_794748	5	110612393	3,699.04	1,728.9168	570,884	570,073 / 99.86%	5 / 251 / 194.27	283,038 / 99.16%	0 / 0%	548,456 / 96.07%	89.14%	17,902 / 3.14%
EPI_ISL_794749	3	115186781	3,852.0142	1,702.4226	569,132	568,528 / 99.89%	8 / 251 / 203.18	277,386 / 97.48%	0 / 0%	544,125 / 95.61%	89.21%	21,209 / 3.73%
EPI_ISL_794750	3	132348222	4,425.9179	2,120.68	686,904	685,592 / 99.81%	5 / 251 / 193.26	336,988 / 98.12%	0 / 0%	661,492 / 96.3%	89.82%	30,992 / 4.51%
EPI_ISL_794751	2	112371803	3,757.8772	1,853.6234	585,444	584,394 / 99.82%	5 / 251 / 192.51	286,838 / 97.99%	0 / 0%	561,078 / 95.84%	88.92%	24,584 / 4.2%
EPI_ISL_794752	44	80513489	2,692.4887	2,288.7892	421,672	394,556 / 93.57%	5 / 251 / 200.23	196,019 / 92.97%	0 / 0%	377,050 / 89.42%	86.83%	12,377 / 2.94%
EPI_ISL_794753	10	128038498	4,281.7944	3,927.0888	651,166	628,852 / 96.57%	5 / 251 / 201.93	311,591 / 95.7%	0 / 0%	609,468 / 93.6%	88.35%	19,992 / 3.07%
EPI_ISL_794754	0	100102098	3,347.5604	1,212.6929	465,456	464,997 / 99.9%	8 / 251 / 215.67	221,532 / 95.19%	0 / 0%	440,286 / 94.59%	87.77%	14,388 / 3.09%
EPI_ISL_794755	1	120378267	4,025.6251	1,734.9336	606,454	605,867 / 99.9%	8 / 251 / 199.26	298,114 / 98.31%	0 / 0%	582,112 / 95.99%	89.04%	22,753 / 3.75%
EPI_ISL_794756	36	130245228	4,355.5907	3,114.5856	724,098	718,794 / 99.27%	5 / 251 / 181.25	356,592 / 98.49%	0 / 0%	697,477 / 96.32%	90.07%	24,646 / 3.4%
EPI_ISL_794757	46	96238662	3,218.3614	2,977.3646	534,446	514,026 / 96.18%	5 / 251 / 186.07	255,350 / 95.56%	0 / 0%	495,458 / 92.7%	88.61%	17,677 / 3.31%
EPI_ISL_794758	45	121011027	4,046.7855	2,844.0336	664,068	657,145 / 98.96%	5 / 251 / 183.93	325,682 / 98.09%	0 / 0%	636,069 / 95.78%	89.83%	23,775 / 3.58%
EPI_ISL_794759	0	107406535	3,591.8314	1,270.8662	509,182	508,406 / 99.85%	5 / 251 / 211.89	241,187 / 94.74%	0 / 0%	483,284 / 94.91%	88.61%	21,854 / 4.29%
EPI_ISL_794760	0	110331693	3,689.653	1,747.7832	543,294	541,859 / 99.74%	5 / 251 / 204.26	262,052 / 96.47%	0 / 0%	517,975 / 95.34%	88.05%	32,716 / 6.02%
EPI_ISL_794761	9	119547111	3,997.83	1,907.8919	586,146	584,961 / 99.8%	8 / 251 / 204.92	287,373 / 98.06%	0 / 0%	562,163 / 95.91%	88.64%	23,341 / 3.98%
EPI_ISL_794762	2	116249001	3,887.5364	1,766.7421	622,122	620,583 / 99.75%	5 / 251 / 187.79	302,175 / 97.14%	0 / 0%	596,375 / 95.86%	89.17%	28,077 / 4.51%
EPI_ISL_876800	7	141415456	4,729.1394	2,253.6088	733,798	730,982 / 99.62%	5 / 251 / 193.82	362,991 / 98.93%	0 / 0%	708,002 / 96.48%	90.58%	22,331 / 3.04%
EPI_ISL_876801	0	62794959	2,099.9552	9398543	306,238	305,761 / 99.84%	11 / 251 / 205.79	149,202 / 97.44%	0 / 0%	283,997 / 92.74%	84.38%	12,825 / 4.19%
EPI_ISL_876802	11	83002574	2,775.7273	1,211.5646	448,286	447,348 / 99.79%	10 / 251 / 185.84	220,970 / 98.58%	0 / 0%	425,760 / 94.98%	87.91%	16,649 / 3.71%
EPI_ISL_876803	11	60515903	2,023.7402	9017734	307,956	307,486 / 99.85%	15 / 251 / 197.18	152,503 / 99.04%	0 / 0%	288,122 / 93.56%	86.36%	10,182 / 3.31%
EPI_ISL_794763	11	123218346	4,120.6015	2,265.169	622,51	613,621 / 98.57%	7 / 251 / 200.58	299,293 / 96.16%	0 / 0%	590,450 / 94.85%	87.92%	38,263 / 6.15%
EPI_ISL_794764	1	124888464	4,176.4527	1,999.2059	637,91	636,321 / 99.75%	5 / 251 / 196.75	310,704 / 97.41%	0 / 0%	612,521 / 96.02%	88.84%	35,775 / 5.61%
EPI_ISL_794765	4	118544954	3,964.3164	2,219.9182	614,376	608,256 / 99%	5 / 251 / 195.23	301,963 / 98.3%	0 / 0%	586,547 / 95.47%	89.64%	24,561 / 4%
EPI_ISL_794766	11	155076751	5,185.9931	2,536.4867	785,56	780,202 / 99.32%	5 / 251 / 199.08	386,849 / 98.49%	0 / 0%	757,025 / 96.37%	90.4%	28,707 / 3.65%
EPI_ISL_794767	13	112038571	3,746.7335	1,711.1066	556,978	556,049 / 99.83%	6 / 251 / 202.15	275,372 / 98.88%	0 / 0%	533,908 / 95.86%	88.92%	22,636 / 4.06%
EPI_ISL_794768	0	72645615	2,429.3755	1,177.6498	356,404	355,912 / 99.86%	5 / 251 / 204.85	170,895 / 95.9%	0 / 0%	333,725 / 93.64%	86.03%	14,344 / 4.02%
EPI_ISL_794769	4	91427817	3,057.4798	1,536.2964	449,098	447,811 / 99.71%	8 / 251 / 204.49	215,470 / 95.96%	0 / 0%	424,647 / 94.56%	86.57%	23,107 / 5.15%
EPI_ISL_794770	7	136244399	4,556.2117	2,137.6577	701,422	696,718 / 99.33%	5 / 251 / 195.73	339,371 / 96.77%	0 / 0%	672,276 / 95.84%	89.31%	43,116 / 6.15%
EPI_ISL_876804	2	75530952	2,525.8654	1,193.4897	382,264	381,332 / 99.76%	9 / 251 / 198.5	183,685 / 96.1%	0 / 0%	359,391 / 94.02%	86.09%	18,468 / 4.83%
EPI_ISL_876805	35	94442437	3,158.293	1,446.8679	531,888	530,341 / 99.71%	8 / 251 / 178.41	261,465 / 98.32%	0 / 0%	507,874 / 95.49%	88.77%	21,646 / 4.07%
EPI_ISL_876806	10	80137079	2,679.901	1,250.9622	414,626	412,697 / 99.53%	7 / 251 / 194.31	199,183 / 96.08%	0 / 0%	389,670 / 93.98%	86.24%	27,337 / 6.59%
EPI_ISL_876807	172	79374708	2,654.4062	1,260.3413	405,078	403,905 / 99.71%	10 / 251 / 197.27	196,319 / 96.93%	0 / 0%	381,390 / 94.15%	86.39%	24,006 / 5.93%
EPI_ISL_794771	26	131673089	4,403.3404	1,998.6609	675,376	672,243 / 99.54%	5 / 251 / 195.96	332,172 / 98.37%	0 / 0%	649,123 / 96.11%	89.39%	26,447 / 3.92%
EPI_ISL_794772	16	126342164	4,225.0665	2,320.4968	679,06	677,775 / 99.81%	7 / 251 / 186.59	334,917 / 98.64%	0 / 0%	655,159 / 96.48%	89.78%	24,194 / 3.56%
EPI_ISL_794773	2	115367462	3,858.0564	1,971.0507	561,63	560,745 / 99.84%	5 / 251 / 206.2	271,754 / 96.77%	0 / 0%	537,020 / 95.62%	88.03%	23,555 / 4.19%
EPI_ISL_794774	0	112545581	3,763.6886	1,921.4665	544,64	542,789 / 99.66%	5 / 251 / 207.6	256,659 / 94.25%	0 / 0%	518,547 / 95.21%	88.05%	29,441 / 5.41%
EPI_ISL_794775	0	77950952	2,606.7937	1,217.0779	396,726	396,158 / 99.86%	5 / 251 / 197.22	195,606 / 98.61%	0 / 0%	375,238 / 94.58%	87.22%	14,593 / 3.68%
EPI_ISL_794776	2	111689478	3,735.0593	2,042.101	581,746	580,565 / 99.8%	7 / 251 / 192.84	280,972 / 96.6%	0 / 0%	557,068 / 95.76%	88.74%	26,482 / 4.55%
EPI_ISL_794777	0	103154555	3,449.639	1,872.2872	499,114	497,557 / 99.69%	6 / 251 / 207.6	232,783 / 93.28%	0 / 0%	473,900 / 94.95%	87.48%	25,738 / 5.16%
EPI_ISL_794778	0	142672709	4,771.1838	2,424.8573	701,984	701,196 / 99.89%	5 / 251 / 204.08	341,576 / 97.32%	0 / 0%	677,131 / 96.46%	89.72%	27,965 / 3.98%
EPI_ISL_794779	9	115871423	3,874.9096	2,114.1785	567,326	566,559 / 99.86%	5 / 251 / 204.73	279,853 / 98.66%	0 / 0%	544,438 / 95.97%	88.43%	17,612 / 3.1%
EPI_ISL_794780	3	126680350	4,236.3759	1,976.8749	625,084	624,312 / 99.88%	5 / 251 / 203.29	308,557 / 98.72%	0 / 0%	601,773 / 96.27%	88.95%	20,856 / 3.34%
EPI_ISL_794781	11	109272609	3,654.2357	1,711.2774	530,66	529,389 / 99.76%	8 / 251 / 206.64	254,775 / 96.02%	0 / 0%	505,290 / 95.22%	87.92%	23,812 / 4.49%
EPI_ISL_794782	12	118906676	3,976.4129	1,731.3692	579,094	578,459 / 99.89%	5 / 251 / 205.9	283,094 / 97.77%	0 / 0%	554,576 / 95.77%	88.42%	19,269 / 3.33%
EPI_ISL_794783	4	131717385	4,404.8218	2,335.7892	655,144	654,167 / 99.85%	5 / 251 / 202.01	319,757 / 97.61%	0 / 0%	630,444 / 96.23%	89.31%	29,093 / 4.44%
EPI_ISL_794784	1	99662445	3,332.8577	1,780.263	481,788	481,018 / 99.84%	7 / 251 / 207.63	234,213 / 97.23%	0 / 0%	458,018 / 95.07%	87.19%	22,541 / 4.68%
EPI_ISL_794785	1	94347144	3,155.1063	1,419.2305	458,362	457,760 / 99.87%	8 / 251 / 206.46	222,536 / 97.1%	0 / 0%	434,547 / 94.8%	87.24%	17,573 / 3.83%
EPI_ISL_794786	23	128788512	4,306.876	2,024.4756	691,09	688,493 / 99.62%	5 / 251 / 187.26	339,815 / 98.34%	0 / 0%	664,967 / 96.22%	89.94%	29,155 / 4.22%
EPI_ISL_794787	10	109096909	3,648.36	1,832.6611	543,236	542,472 / 99.86%	5 / 251 / 201.51	264,438 / 97.36%	0 / 0%	519,527 / 95.64%	88.15%	19,480 / 3.59%
EPI_ISL_794788	4	105544876	3,529.5748	1,590.6377	542,336	541,625 / 99.87%	5 / 251 / 195.2	268,400 / 98.98%	0 / 0%	519,554 / 95.8%	88.72%	18,120 / 3.34%
EPI_ISL_794789	11	106749629	3,569.8635	1,695.5389	545,494	544,644 / 99.84%	5 / 251 / 196.35	269,850 / 98.94%	0 / 0%	522,686 / 95.82%	88.91%	18,164 / 3.33%
EPI_ISL_794790	16	117199637	3,919.3271	1,926.8275	585,814	584,070 / 99.7%	5 / 251 / 200.82	289,936 / 98.99%	0 / 0%	562,720 / 96.06%	88.84%	19,081 / 3.26%
EPI_ISL_794791	12	103005515	3,444.6549	1,649.5709	505,568	504,611 / 99.81%	8 / 251 / 204.32	249,852 / 98.84%	0 / 0%	483,164 / 95.57%	87.91%	17,067 / 3.38%
EPI_ISL_794792	59	110531175	3,696.3239	1,725.1119	553,45	552,242 / 99.78%	6 / 251 / 200.4	271,831 / 98.23%	0 / 0%	529,619 / 95.69%	88.1%	25,107 / 4.54%
EPI_ISL_794793	4	105160868	3,516.733	1,746.3033	545,976	541,204 / 99.13%	5 / 251 / 194.31	261,516 / 95.8%	0 / 0%	518,097 / 94.89%	87.95%	24,777 / 4.54%
EPI_ISL_794794	29	105196006	3,517.9081	1,644.398	544,88	542,287 / 99.52%	5 / 251 / 193.98	264,411 / 97.05%	0 / 0%	518,993 / 95.25%	87.61%	31,544 / 5.79%
EPI_ISL_794795	2	79260647	2,650.5918	1,217.4526	405,974	405,466 / 99.87%	5 / 251 / 195.95	199,835 / 98.45%	0 / 0%	384,194 / 94.64%	87.13%	15,841 / 3.9%

EPI_ISL_794796	0	65336866	2,184.9602	1,302.5088	334,384	333,789 / 99.82%	5 / 251 / 196.4	151,773 / 90.78%	0 / 0%	312,239 / 93.38%	85.49%	14,446 / 4.32%
EPI_ISL_794797	2	96301490	3,220.4625	1,608.3298	473,222	472,456 / 99.84%	5 / 251 / 204.18	230,208 / 97.29%	0 / 0%	450,055 / 95.1%	87.32%	17,720 / 3.74%
EPI_ISL_794798	10	96039693	3,211.7076	1,451.4985	543,642	542,519 / 99.79%	5 / 251 / 177.23	268,623 / 98.82%	0 / 0%	520,240 / 95.7%	89.02%	19,355 / 3.56%
EPI_ISL_794799	0	85443163	2,857.3442	1,411.3961	425,222	424,622 / 99.86%	5 / 251 / 201.6	208,427 / 98.03%	0 / 0%	403,051 / 94.79%	86.83%	17,469 / 4.11%
EPI_ISL_794800	16	88643373	2,964.3639	1,342.1671	444,79	443,764 / 99.77%	5 / 251 / 199.92	218,640 / 98.31%	0 / 0%	422,211 / 94.92%	87.66%	18,496 / 4.16%
EPI_ISL_794801	4	105983428	3,544.2406	1,663.1775	526,554	525,638 / 99.83%	7 / 251 / 201.91	259,096 / 98.41%	0 / 0%	503,646 / 95.65%	88.03%	22,034 / 4.18%
EPI_ISL_794802	4	80005869	2,675.5131	1,375.6334	394,742	394,106 / 99.84%	5 / 251 / 203.33	190,555 / 96.55%	0 / 0%	371,881 / 94.21%	86.37%	14,800 / 3.75%
EPI_ISL_794803	16	117229652	3,920.3308	1,802.4557	600,972	596,693 / 99.29%	5 / 251 / 196.48	295,449 / 98.32%	0 / 0%	574,309 / 95.56%	89.03%	19,899 / 3.31%
EPI_ISL_794804	10	97454234	3,259.0119	1,503.9186	500,934	499,911 / 99.8%	9 / 251 / 195.15	246,918 / 98.58%	0 / 0%	478,177 / 95.46%	88.27%	20,288 / 4.05%
EPI_ISL_794805	36	107069611	3,580.5642	1,754.111	547,532	545,407 / 99.61%	5 / 251 / 196.39	271,094 / 99.02%	0 / 0%	524,281 / 95.75%	89.3%	16,113 / 2.94%
EPI_ISL_794806	6	77221229	2,582.3907	1,013.1293	375,398	374,864 / 99.86%	7 / 251 / 206.2	179,581 / 95.67%	0 / 0%	351,767 / 93.71%	86.07%	12,426 / 3.31%
EPI_ISL_794807	7	106810071	3,571.8848	1,669.4096	551,082	549,723 / 99.75%	5 / 251 / 194.52	270,118 / 98.03%	0 / 0%	526,880 / 95.61%	88.21%	23,295 / 4.23%
EPI_ISL_794808	44	111824322	3,739.5687	1,737.782	595,99	594,191 / 99.7%	5 / 251 / 188.31	293,164 / 98.38%	0 / 0%	571,814 / 95.94%	89.36%	23,280 / 3.91%
EPI_ISL_794809	2	39806155	1,331.176	6245717	427,186	426,319 / 99.8%	5 / 251 / 184.62	210,065 / 98.35%	0 / 0%	404,879 / 94.78%	87.38%	17,946 / 4.2%
EPI_ISL_794810	1	66779117	2,233.1912	9942675	322,76	322,200 / 99.83%	6 / 251 / 207.58	154,263 / 95.59%	0 / 0%	300,033 / 92.96%	84.8%	12,410 / 3.84%
EPI_ISL_794811	2	74485142	2,490.892	1,253.5352	361,874	361,033 / 99.77%	5 / 251 / 206.59	172,826 / 95.52%	0 / 0%	338,403 / 93.51%	84.98%	17,341 / 4.79%
EPI_ISL_794812	1	108957249	3,643.6896	1,971.7603	546,56	545,848 / 99.87%	7 / 251 / 200.15	264,406 / 96.75%	0 / 0%	522,547 / 95.61%	88.37%	21,794 / 3.99%
EPI_ISL_794813	4	95380175	3,189.6524	1,489.1008	477,618	475,923 / 99.65%	10 / 251 / 200.75	234,171 / 98.06%	0 / 0%	453,885 / 95.03%	87.92%	22,831 / 4.78%
EPI_ISL_794814	4	86701964	2,899.4403	1,326.2454	431,922	430,728 / 99.72%	6 / 251 / 201.59	211,709 / 98.03%	0 / 0%	409,001 / 94.69%	86.73%	20,730 / 4.8%
EPI_ISL_794815	25	102828384	3,438.7314	1,667.914	537,894	536,355 / 99.71%	7 / 251 / 191.87	264,549 / 98.36%	0 / 0%	514,081 / 95.57%	88.72%	20,740 / 3.86%
EPI_ISL_794816	9	130048288	4,349.0047	2,043.9621	662,756	661,635 / 99.83%	8 / 251 / 196.79	327,185 / 98.73%	0 / 0%	639,038 / 96.42%	89.53%	21,480 / 3.24%
EPI_ISL_794817	4	77056828	2,576.8929	1,259.3559	396,872	394,482 / 99.4%	8 / 251 / 195.2	184,880 / 93.17%	0 / 0%	370,877 / 93.45%	86.05%	23,858 / 6.01%
EPI_ISL_876742	12	70978152	2,373.6131	1,105.2828	355,69	354,402 / 99.64%	8 / 251 / 200.79	173,602 / 97.61%	0 / 0%	332,987 / 93.62%	85.96%	12,949 / 3.64%
EPI_ISL_918408	37	91181766	3,049.2514	1,484.0657	466,798	464,317 / 99.47%	10 / 251 / 196.4	229,490 / 98.33%	0 / 0%	443,306 / 94.97%	87.79%	23,814 / 5.1%
EPI_ISL_918409	17	85647078	2,864.1634	1,267.6296	443,954	443,346 / 99.86%	10 / 251 / 193.4	219,616 / 98.94%	0 / 0%	422,188 / 95.1%	88.38%	16,332 / 3.68%
EPI_ISL_876743	0	83081326	2,778.3609	1,162.6421	432,844	432,068 / 99.82%	7 / 251 / 192.84	213,392 / 98.6%	0 / 0%	409,990 / 94.72%	87.23%	22,592 / 5.22%
EPI_ISL_100096[illegible]	10	98091632	3,280.3275	1,413.6387	564,73	562,725 / 99.64%	5 / 251 / 174.45	278,643 / 98.68%	0 / 0%	540,390 / 95.69%	89.67%	23,205 / 4.11%
EPI_ISL_876744	15	69550139	2,325.8582	1,073.2041	361,714	358,673 / 99.16%	8 / 251 / 193.54	170,568 / 94.31%	0 / 0%	336,078 / 92.91%	84.79%	27,127 / 7.5%
EPI_ISL_876745	3	112964076	3,777.6837	1,602.6885	557,228	555,830 / 99.75%	9 / 251 / 203.48	268,314 / 96.3%	0 / 0%	532,221 / 95.51%	88.09%	24,658 / 4.43%
EPI_ISL_876746	2	77256368	2,583.5658	1,178.2588	440,882	439,929 / 99.78%	5 / 251 / 175.93	216,503 / 98.21%	0 / 0%	418,118 / 94.84%	87.81%	19,733 / 4.48%
EPI_ISL_876747	0	85247606	2,850.8045	1,280.0498	434,96	434,395 / 99.87%	13 / 251 / 196.71	215,002 / 98.86%	0 / 0%	413,321 / 95.03%	87.96%	13,738 / 3.16%
EPI_ISL_876787	0	51490795	1,721.9274	8578938	276,262	275,567 / 99.75%	5 / 251 / 187.42	118,488 / 85.78%	0 / 0%	254,459 / 92.11%	84.5%	11,305 / 4.09%
EPI_ISL_876788	4	61822156	2,067.4232	9621756	345,172	344,090 / 99.69%	8 / 251 / 179.99	163,304 / 94.62%	0 / 0%	322,918 / 93.55%	85.58%	15,532 / 4.5%
EPI_ISL_876795	2	66164935	2,212.6521	1,107.1303	382,342	381,214 / 99.7%	8 / 251 / 173.92	179,056 / 93.66%	0 / 0%	359,082 / 93.92%	86.99%	16,505 / 4.32%
EPI_ISL_876789	4	65773366	2,199.5574	1,179.2296	336,292	335,573 / 99.79%	12 / 251 / 196.48	152,286 / 90.57%	0 / 0%	314,010 / 93.37%	85.33%	13,608 / 4.05%
EPI_ISL_876796	3	84636056	2,830.3533	1,274.6018	428,316	427,695 / 99.86%	6 / 251 / 198.44	208,858 / 97.53%	0 / 0%	405,604 / 94.7%	87.48%	17,597 / 4.11%
EPI_ISL_876790	2	85024124	2,843.3309	1,427.4807	454,918	451,604 / 99.27%	5 / 251 / 188.53	221,413 / 97.34%	0 / 0%	430,068 / 94.54%	87.43%	17,164 / 3.77%
EPI_ISL_876797	4	83199902	2,782.3263	1,265.3217	470,19	469,293 / 99.81%	5 / 251 / 177.59	229,541 / 97.64%	0 / 0%	447,852 / 95.25%	88.28%	13,744 / 2.92%
EPI_ISL_876791	4	82452524	2,757.3328	1,273.1093	449,146	447,636 / 99.66%	15 / 251 / 184.59	217,373 / 96.79%	0 / 0%	425,984 / 94.84%	87.6%	16,163 / 3.6%
EPI_ISL_876798	38	90679790	3,032.4646	1,486.83	523,458	520,049 / 99.35%	7 / 251 / 174.46	255,607 / 97.66%	0 / 0%	497,827 / 95.1%	0,89	20,856 / 3.98%
EPI_ISL_876799	9	64220518	2,147.6279	1,091.8259	348,174	346,600 / 99.55%	8 / 251 / 185.46	158,938 / 91.3%	0 / 0%	325,045 / 93.36%	85.7%	17,265 / 4.96%
EPI_ISL_876792	4	84866882	2,838.0725	1,359.0901	507,308	506,080 / 99.76%	10 / 251 / 168.02	248,617 / 98.01%	0 / 0%	483,843 / 95.37%	88.86%	20,726 / 4.09%
EPI_ISL_876793	0	69754278	2,332.6849	1,064.739	401,716	398,328 / 99.16%	7 / 251 / 175.22	187,337 / 93.27%	0 / 0%	375,652 / 93.51%	86.46%	27,213 / 6.77%
EPI_ISL_876776	4	76727106	2,565.8665	1,204.3899	384,952	383,127 / 99.53%	5 / 251 / 200.42	189,504 / 98.46%	0 / 0%	362,723 / 94.23%	87.11%	12,953 / 3.36%
EPI_ISL_876778	7	98054574	3,279.0882	1,552.9455	490,132	489,553 / 99.88%	5 / 251 / 200.61	240,728 / 98.23%	0 / 0%	467,249 / 95.33%	88.15%	15,348 / 3.13%
EPI_ISL_876777	4	84933802	2,840.3104	1,327.3629	426,592	425,857 / 99.83%	5 / 251 / 199.73	211,263 / 99.05%	0 / 0%	405,385 / 95.03%	87.64%	13,579 / 3.18%
EPI_ISL_876808	2	92350706	3,088.3425	1,376.767	466,944	465,655 / 99.72%	5 / 251 / 198.48	227,606 / 97.49%	0 / 0%	443,374 / 94.95%	87.01%	25,150 / 5.39%
EPI_ISL_876809	4	78821878	2,635.9187	1,220.0789	388,332	387,822 / 99.87%	8 / 251 / 203.5	189,985 / 97.85%	0 / 0%	366,520 / 94.38%	86.56%	12,913 / 3.33%
EPI_ISL_876784	11	100131854	3,348.5555	1,428.756	567,394	566,162 / 99.78%	10 / 251 / 177.14	279,158 / 98.4%	0 / 0%	543,254 / 95.75%	89.02%	23,090 / 4.07%
EPI_ISL_876785	0	76265492	2,550.4295	1,128.0113	394,572	392,540 / 99.49%	8 / 251 / 194.4	189,197 / 95.9%	0 / 0%	369,795 / 93.72%	85.9%	25,505 / 6.46%
EPI_ISL_876794	32	83935879	2,806.9384	1,222.9885	422,222	420,995 / 99.71%	5 / 251 / 199.8	205,315 / 97.25%	0 / 0%	398,228 / 94.32%	86.38%	25,254 / 5.98%
EPI_ISL_876786	2	77903215	2,605.1973	1,386.1186	384,286	383,671 / 99.84%	5 / 251 / 203.67	184,948 / 96.26%	0 / 0%	361,499 / 94.07%	86.19%	15,397 / 4.01%
EPI_ISL_876748	7	112542925	3,763.5998	1,793.5816	578,812	577,912 / 99.84%	8 / 251 / 195.2	283,523 / 97.97%	0 / 0%	554,655 / 95.83%	88.94%	20,649 / 3.57%
EPI_ISL_876749	3	112766910	3,771.0902	1,814.9289	580,38	579,406 / 99.83%	5 / 251 / 195.03	286,060 / 98.58%	0 / 0%	556,864 / 95.95%	89.37%	19,802 / 3.41%
EPI_ISL_876751	3	95037092	3,178.1792	1,385.6336	513,734	512,962 / 99.85%	5 / 251 / 185.86	255,077 / 99.3%	0 / 0%	491,301 / 95.63%	89.62%	18,439 / 3.59%
EPI_ISL_876750	0	104394657	3,491.1098	1,741.6496	528,142	526,752 / 99.74%	7 / 251 / 198.8	256,518 / 97.14%	0 / 0%	503,116 / 95.26%	87.88%	30,786 / 5.83%
EPI_ISL_876779	5	118490613	3,962.4992	1,749.5261	614,994	612,376 / 99.57%	5 / 251 / 193.69	298,724 / 97.15%	0 / 0%	588,369 / 95.67%	88.57%	39,088 / 6.36%
EPI_ISL_876780	4	105925135	3,542.2912	1,557.0157	538,202	537,250 / 99.82%	10 / 251 / 197.65	265,119 / 98.52%	0 / 0%	514,690 / 95.63%	88.5%	24,540 / 4.56%
EPI_ISL_876781	2	80851628	2,703.7965	1,338.0156	446,012	444,803 / 99.73%	5 / 251 / 182.22	215,054 / 96.43%	0 / 0%	421,617 / 94.53%	87.41%	22,618 / 5.07%
EPI_ISL_876782	0	116705276	3,902.7949	1,792.9653	591,438	590,596 / 99.86%	5 / 251 / 198.13	290,601 / 98.27%	0 / 0%	567,254 / 95.91%	88.9%	
EPI_ISL_876783	5	84985975	2,842.0551	1,394.3809	426,598	422,596 / 99.06%	5 / 251 / 201.06	207,562 / 97.31%	0 / 0%	401,232 / 94.05%	86.5%	21,915 / 5.14%
EPI_ISL_876760	4	122105042	4,083.371	1,877.2895	649,586	648,549 / 99.84%	10 / 251 / 188.65	318,598 / 98.09%	0 / 0%	624,784 / 96.18%	89.95%	22,650 / 3.49%
EPI_ISL_876752	4	88922079	2,973.6842	1,322.5839	511,454	510,351 / 99.78%	10 / 251 / 174.65	252,293 / 98.66%	0 / 0%	488,184 / 95.45%	89.31%	20,383 / 3.99%
EPI_ISL_876761	0	78253005	2,616.8948	1,205.5417	392,084	390,956 / 99.71%	5 / 251 / 200.52	188,187 / 95.99%	0 / 0%	367,758 / 93.8%	86.55%	21,565 / 5.5%
EPI_ISL_876762	2	98775475	3,303.1962	1,759.2575	489,852	489,187 / 99.86%	5 / 251 / 202.4	239,129 / 97.63%	0 / 0%	466,622 / 95.26%	87.91%	17,823 / 3.64%
EPI_ISL_876753	36	123342464	4,124.7522	1,770.3059	648,564	647,461 / 99.83%	5 / 251 / 190.83	321,791 / 99.23%	0 / 0%	624,978 / 96.36%	90.35%	19,182 / 2.96%
EPI_ISL_876740	2	82475624	2,758.1053	1,255.5804	424,738	424,201 / 99.87%	5 / 251 / 194.82	208,974 / 98.4%	0 / 0%	402,089 / 94.67%	87.65%	14,146 / 3.33%
EPI_ISL_876763	4	105595723	3,531.2752	1,550.5864	513,074	511,825 / 99.76%	5 / 251 / 206.61	247,667 / 96.54%	0 / 0%	487,170 / 94.95%	88.13%	25,537 / 4.98%
EPI_ISL_876754	9	119737700	4,004.2036	1,839.5719	636,83	635,832 / 99.84%	5 / 251 / 188.97	313,924 / 98.59%	0 / 0%	612,449 / 96.17%	90.03%	24,054 / 3.78%
EPI_ISL_876764	2	96756428	3,235.6763	1,435.5376	472,822	471,948 / 99.82%	12 / 251 / 205.38	231,186 / 97.79%	0 / 0%	449,381 / 95.04%	87.36%	19,537 / 4.13%
EPI_ISL_876755	17	113333618	3,790.0417	1,693.6638	570,504	569,614 / 99.84%	10 / 251 / 199.37	282,162 / 98.92%	0 / 0%	547,412 / 95.95%	89.15%	21,071 / 3.69%
EPI_ISL_876756	4	104571336	3,497.0182	1,594.6396	574,966	573,568 / 99.76%	5 / 251 / 182.67	282,483 / 98.26%	0 / 0%	550,689 / 95.78%	89.4%	27,147 / 4.72%
EPI_ISL_876765	14	96197206	3,216.9751	1,435.0992	488,772	487,947 / 99.83%	5 / 251 / 197.5	239,408 / 97.96%	0 / 0%	464,970 / 95.13%	88.53%	16,418 / 3.36%
EPI_ISL_876766	10	117920645	3,943.4386	1,742.7128	591,992	590,826 / 99.8%	5 / 251 / 199.92	293,172 / 99.05%	0 / 0%	568,668 / 96.06%	88.96%	18,281 / 3.09%
EPI_ISL_876767	4	85022675	2,843.2824	1,203.8143	429,982	429,439 / 99.87%	5 / 251 / 198.45	211,507 / 98.38%	0 / 0%	407,725 / 94.82%	87.9%	14,344 / 3.34%
EPI_ISL_876768	4	117357888	3,924.6192	1,768.3748	591,414	590,455 / 99.84%	6 / 251 / 199.12	290,410 / 98.21%	0 / 0%	567,329 / 95.93%	89.06%	20,069 / 3.39%
EPI_ISL_876769	0	92140708	3,081.3199	1,451.9804	450,668	449,841 / 99.82%	6 / 251 / 205.28	222,402 / 98.7%	0 / 0%	428,650 / 95.11%	87.2%	16,687 / 3.7%

EPI_ISL_876757	2	89377944	2,988.929	1,504.6834	446,08	444,917 / 99.74%	10 / 251 / 201.46	214,837 / 96.32%	0 / 0%	421,825 / 94.56%	87.07%	24,603 / 5.52%
EPI_ISL_876770	3	150458441	5,031.55	2,162.8569	745,226	743,313 / 99.74%	7 / 251 / 202.7	357,804 / 96.03%	0 / 0%	718,382 / 96.4%	90.1%	33,568 / 4.5%
EPI_ISL_876771	2	105463178	3,526.8427	1,508.8757	535,4	532,270 / 99.42%	5 / 251 / 198.03	256,105 / 95.67%	0 / 0%	507,765 / 94.84%	87.97%	37,701 / 7.04%
EPI_ISL_876772	8	107274543	3,587.4174	1,485.7044	553,246	552,507 / 99.87%	5 / 251 / 194.58	274,310 / 99.16%	0 / 0%	530,483 / 95.89%	89.46%	18,378 / 3.32%
EPI_ISL_876773	8	85632356	2,863.6711	1,183.9386	438,514	437,827 / 99.84%	5 / 251 / 195.86	217,240 / 99.08%	0 / 0%	416,692 / 95.02%	88.34%	12,887 / 2.94%
EPI_ISL_876774	0	90287817	3,019.3565	1,608.229	443,864	443,163 / 99.84%	5 / 251 / 204.1	217,139 / 97.84%	0 / 0%	420,913 / 94.83%	87.12%	15,046 / 3.39%
EPI_ISL_876758	3	104557618	3,496.5595	1,558.3416	582,524	581,334 / 99.8%	5 / 251 / 180.07	288,006 / 98.88%	0 / 0%	558,940 / 95.95%	89.81%	21,378 / 3.67%
EPI_ISL_876775	4	108456489	3,626.9434	1,654.9108	533,242	532,024 / 99.77%	5 / 251 / 204.15	262,098 / 98.3%	0 / 0%	509,679 / 95.58%	87.79%	20,100 / 3.77%
EPI_ISL_876759	0	42514933	1,421.7615	7550389	201,272	200,468 / 99.6%	10 / 251 / 212.29	91,352 / 90.77%	0 / 0%	180,473 / 89.67%	80.46%	10,450 / 5.19%
EPI_ISL_876810	22	99009916	3,311.0362	1,482.0016	500,304	499,077 / 99.75%	5 / 251 / 198.66	247,599 / 98.98%	0 / 0%	477,636 / 95.47%	89.04%	15,490 / 3.1%
EPI_ISL_876741	1	120745113	4,037.893	2,328.2373	599,502	591,685 / 98.7%	9 / 251 / 204	292,786 / 97.68%	0 / 0%	569,923 / 95.07%	88.96%	24,613 / 4.11%
EPI_ISL_876738	127	36294991	1,213.7575	1,541.217	310,248	190,508 / 61.41%	5 / 251 / 174.65	93,659 / 60.38%	0 / 0%	177,481 / 57.21%	81.95%	12,077 / 3.89%
EPI_ISL_722861	30	93153980	3,115.2052	2,256.0632	548,230	518,491 / 94.58%	13 / 236 / 180.32	255,886 / 93.35%	0 / 0%	493,817 / 90.07%	85.98%	19,715 / 3.6%

Table S2: Quality(raw sequencing)data for the 144 SARS-CoV-2 genomes sequenced in this study.

Nextstrain Clade	***Pangolin lineage***	***N. of genomes***	***% of genomes***
**20E (EU1)**	B.1.177	177	97,79
	B.1.177.8	2	1,10
	B.1.178	1	0,55
	B.1.179	1	0,55
**20B**	B.1.1.229	45	43,69
	B.1.1.74	27	26,21
	B.1.1.277	7	6,80
	B.1.1.305	4	3,88
	B.1.1.207	3	2,91
	B.1.1.39	3	2,91
	B.1	2	1,94
	B.1.1.316	2	1,94
	B.1.1.1	1	0,97
	B.1.1.115	1	0,97
	B.1.1.162	1	0,97
	B.1.1.204	1	0,97
	B.1.1.220	1	0,97
	B.1.1.265	1	0,97
	B.1.1.288	1	0,97
	B.1.1.293	1	0,97
	B.1.1.317	1	0,97
	B.1.1.33	1	0,97
**20A**	B.1.416	14	41,18
	B.1	9	26,47
	B.1.258	3	8,82
	B.1.258.14	3	8,82
	B.1.179	2	5,88
	B.1.221	1	2,94
	B.1.36	1	2,94

	B.1.389	1	2,94
**20A.EU2**	B.1.160	20	100,00
**20I/501Y.V1**	B.1.1.7	9	100,00
**20D**	B.1.1.1	6	100

Table S3: Pangolin lineage prediction for the 353 SARS-CoV-2 genome sequences from Apulia and Basilicata, surveyed in this study. The percentages refer to the frequency compared to the total number of genomes assigned to the same clade.

Nextstrain Clade	***N. of Italian genomes***	***% of Italian genomes***	***N. of Apulian genomes***	***% of Apulian genomes***
**19A**	21	0,94	0	0,00
**19B**	15	0,67	0	0,00
**20A**	692	30,84	34	9,63
**20A.EU2**	81	3,61	20	5,67
**20B**	596	26,56	103	29,18
**20C**	13	0,58	0	0,00
**20D**	102	4,55	6	1,70
**20E (EU1)**	714	31,82	181	51,27
**20I/501Y.V1**	10	0,45	9	2,55
Total genomes	2244	100,00	353	100,00

Table S4: Nextclade prediction for the 2244 Italian sequences of the SARS-CoV-2 genome and the 353 sequences from Apulia and Basilicata, with the corresponding incidence rates.
